# Multifunctional CdS Semiconductor Nanocrystals: Enhanced Photocatalytic and Supercapacitor Performance with DFT Insights

**DOI:** 10.3390/nano16211406

**Published:** 2026-11-01

**Authors:** Sidhaling B. Bhairunagi, Ketankumar A. Ganure, Neha Kondal, Rekha M. Ovhal, Vishwajeet V. Aherkar, Laxman A. Dhale, R. Saidur, Sharanabasava V. Ganachari

**Affiliations:** 1 Department of Chemistry, C. B. Khedgi’s Basaveshwar Science Raja Vijaysinh Commerce & Raja Jaysinh Arts College, Akkalkot 413216, Maharashtra, India; sbhairunagi@gmail.com (S.B.B.); laxman.dhale@gmail.com (L.A.D.); 2 Department of Chemistry, Dr. D. Y. Patil Institute of Technology, Sant Tukaram Nagar, Pimpri, Pune 411018, Maharashtra, India; 3 School of Technology and Research, Dr. D. Y. Patil Unitech Society, Dnyaan Prasad Global University (DPGU), Sant Tukaram Nagar, Pimpri, Pune 411018, Maharashtra, India; 4 Department of Physics, Centre for Research and Development, Chandigarh University, Mohali 140413, Punjab, India; nehakondal91@gmail.com; 5 Department of Chemistry, Walchand College of Arts and Science (Autonomous), Solapur 413006, Maharashtra, India; 6 Department of Chemistry, Sangameshwar College (Autonomous), Solapur 413001, Maharashtra, India; aherkar@yahoo.com; 7 Research Centre for Nanomaterials and Energy Technology (RCNMET), Faculty of Engineering and Technology, Sunway University, Bandar Sunway 47500, Selangor Darul Ehsan, Malaysia; saidur@sunway.edu.my; 8 Centre for Energy and Environment, School of Advanced Sciences, KLE Technological University, Hubballi 580031, Karnataka, India

**Keywords:** CdS semiconductor nanocrystals, co-precipitation, photocatalysis, supercapacitor performance, antibacterial activity, density functional theory (DFT)

## Abstract

Cadmium sulphide (CdS) semiconductor nanocrystals are of interest because of their optical properties and potential in photocatalysis. Here, we examine their structural, optical, photocatalytic, electrochemical, antimicrobial, and theoretical characteristics for possible environmental and energy-related applications. CdS nanocrystals were prepared by aqueous co-precipitation. X-ray diffraction (XRD) confirmed the formation of hexagonal wurtzite CdS, while FESEM, HRTEM, and EDAX were used to examine morphology, crystallinity, and elemental composition. UV–visible spectroscopy and Tauc analysis gave an optical band gap of 2.15 eV. Under visible-light irradiation, the material degraded 89.93% of methylene blue within 120 min. Antibacterial activity was also observed against the Gram-positive bacterium *Staphylococcus aureus* and the Gram-negative bacterium *Pseudomonas aeruginosa*. Cyclic voltammetry yielded a specific capacitance of 17.75 F g^−1^, indicating the potential of the material for further investigation in energy storage applications. Density functional theory (DFT) calculations were used to examine the electronic structure, charge distribution, and related features of the CdS nanoclusters. Taken together, the experimental and theoretical results provide an initial assessment of the multifunctional behaviour of the prepared CdS nanocrystals. Further work is needed to establish their performance and safety under application-specific conditions.

## 1. Introduction

The continued growth of industry, transportation, and technology has increased reliance on fossil fuels and intensified concerns about resource depletion and greenhouse-gas emissions. These challenges have encouraged research into materials that can support environmental remediation, energy technologies, and biomedical applications. Nanostructured materials are promising in this context because their high surface-area-to-volume ratios, surface-active sites, and size-dependent effects can influence their physicochemical behaviour [1,2,3,4]. Semiconductor nanomaterials are consequently being investigated for applications in optoelectronics, photocatalysis, sensing, biomedical devices, and electrochemical energy storage.

Among the II–VI semiconductor materials, CdS semiconductor nanocrystals (CdS–SNCs) are notable for their visible-light response, optical absorption, and charge-transport characteristics. CdS has a direct band gap of approximately 2.15 eV, making it relevant to visible-light-driven photocatalytic and photoelectrochemical processes [5,6,7,8]. Depending on their morphology, including quantum dots, nanospheres, nanorods, nanoflakes, and hierarchical structures, CdS materials can exhibit different light-absorption behaviour, surface activity, charge separation, and interfacial charge transfer kinetics.

Considerable effort has been directed towards making nanomaterial synthesis more sustainable. Established approaches, including hydrothermal, sol–gel, microwave-assisted, chemical-bath deposition, and hot-injection methods, can involve high temperatures or pressures, hazardous reagents, or demanding purification steps [9,10,11]. Aqueous co-precipitation is attractive because it is relatively simple, economical, and potentially scalable, with modest energy requirements. Nevertheless, controlling nucleation, aggregation, particle shape, and defects remains challenging. The electrochemical behaviour of co-precipitated CdS nanocrystals, particularly for supercapacitor-related applications, also warrants further investigation.

CdS is a candidate for visible-light photocatalysis because it absorbs light in a useful spectral range and can generate photogenerated charge carriers. Its activity has been investigated for the degradation of organic dyes, including methylene blue and rhodamine B [12,13,14]. Practical limitations remain, however, including photocorrosion, electron–hole recombination, and instability during prolonged illumination. CdS nanocrystals have also been investigated for antibacterial applications, where proposed effects include reactive oxygen species (ROS), damage to cell membranes, and the possible contribution of released cadmium ions. Aggregation, instability in water, and incomplete understanding of the mechanisms continue to limit interpretation of these effects.

Electrochemical energy storage is another potential application of semiconductor nanomaterials. CdS has been explored for pseudocapacitive behaviour associated with redox processes and ion transport, but its use in energy storage devices is constrained by issues such as limited electrical conductivity, structural instability, and particle aggregation [15,16,17]. Much of the existing work has focused on CdS composites or doped materials. More integrated studies of pristine CdS nanocrystals—linking morphology, crystallinity, electronic structure, and performance across several applications—remain useful, particularly when experimental findings are considered alongside density functional theory (DFT) calculations.

These considerations motivate a combined investigation of co-precipitated CdS nanocrystals across photocatalytic, antimicrobial, and electrochemical applications, supported by DFT calculations. In particular, the relationships between material structure, measured properties, and performance need to be assessed without overstating what can be inferred from individual measurements. Such research may contribute to the broader development of nanomaterials relevant to clean water (SDG 6), clean energy (SDG 7), health (SDG 3), and responsible production (SDG 12).

In this study, CdS semiconductor nanocrystals were prepared by aqueous co-precipitation and examined using XRD, FESEM, EDAX, HRTEM, and UV–visible spectroscopy. Their photocatalytic activity was evaluated through methylene-blue degradation under visible-light irradiation. Antimicrobial activity and electrochemical behaviour were also investigated, and DFT calculations were used to examine the electronic structure and charge distribution. These complementary analyses provide a broader picture of the properties of the prepared CdS nanocrystals.

The contribution of this work lies in evaluating pristine wurtzite CdS nanocrystals through a combined experimental and theoretical study rather than in claiming that co-precipitation itself is a new synthesis method. The study brings together visible-light photocatalysis, antimicrobial testing, electrochemical measurements, and DFT analysis to examine the material’s multifunctional properties. This integrated approach supports a structure–property–performance discussion while identifying mechanisms that still require direct experimental verification.

Co-precipitation is an established method for preparing CdS, so the present work does not claim novelty solely based on the synthesis route or a unique structural modification. Instead, it evaluates pristine wurtzite CdS nanocrystals using a coordinated set of structural, optical, photocatalytic, electrochemical, antimicrobial, and DFT analyses. The aim is to provide an integrated experimental and theoretical assessment without asserting superiority over all previously reported CdS-based materials.

## 2. Materials and Methods

### 2.1. Materials

Cadmium chloride monohydrate (CdCl_2_·H_2_O) and sodium sulphide nonahydrate (Na_2_S·9H_2_O) were purchased from Sigma-Aldrich (Pune, India) and used without further purification. All solutions were prepared using deionised water.

### 2.2. Synthesis of CdS Semiconductor Nanocrystals (CdS–SNCs)

CdS–SNCs were synthesised via a simple and environmentally friendly aqueous co-precipitation method using cadmium chloride monohydrate (CdCl_2_·H_2_O) as the cadmium precursor and sodium sulphide (Na_2_S) as the sulphur precursor. Trisodium citrate was employed as a complexing/capping agent. Initially, a 1N aqueous solution of cadmium chloride was prepared, followed by the addition of trisodium citrate to facilitate the formation of a Cd–citrate complex. Subsequently, a 2N Na_2_S solution was added dropwise into the reaction mixture maintained at 50–60 °C under continuous stirring, resulting in the controlled nucleation and growth of CdS semiconductor nanocrystals.

Cd^2+^ + S^2−^ → CdS↓

**Figure 1:**
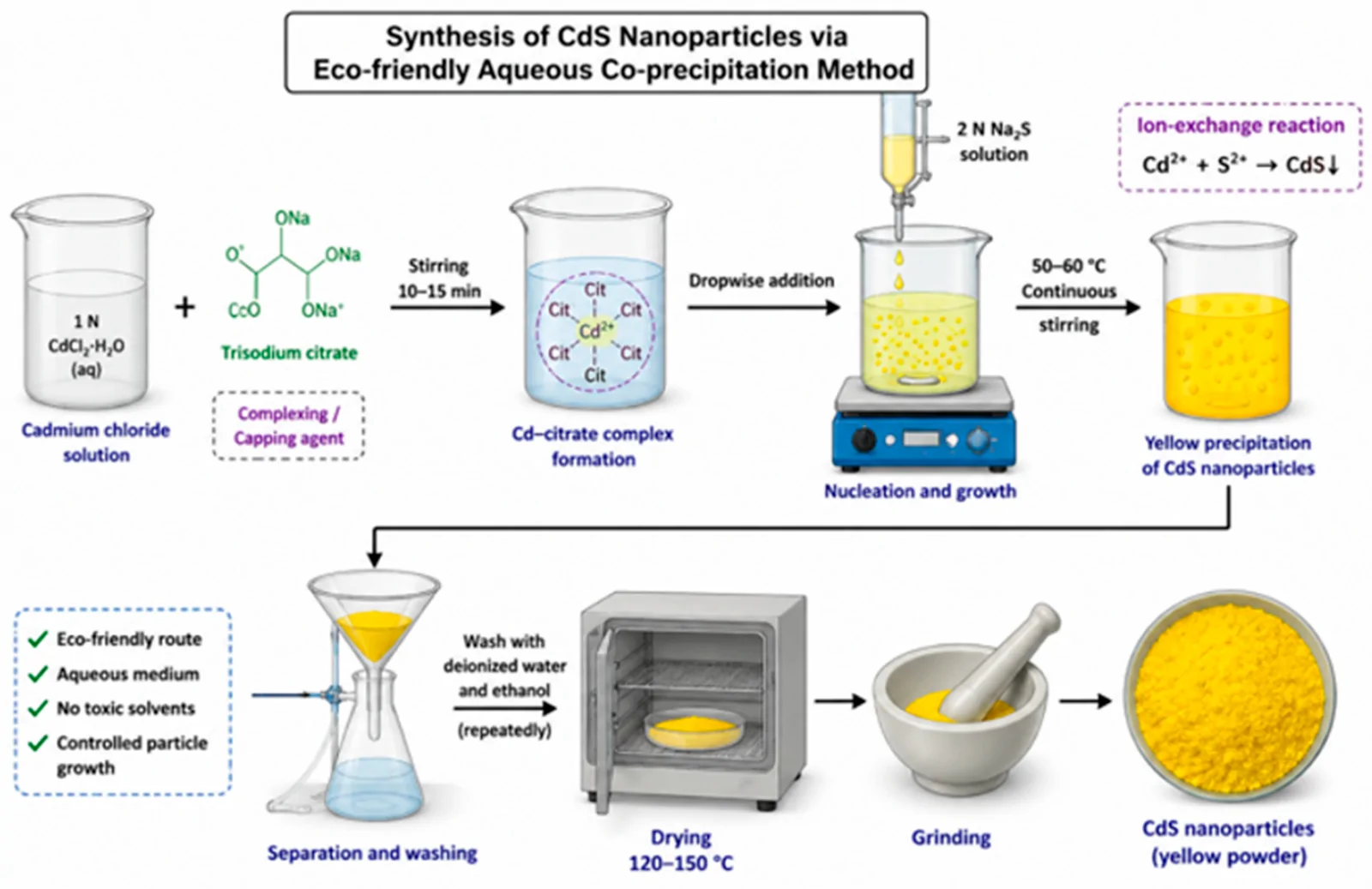
Schematic illustration of the eco-friendly aqueous co-precipitation synthesis of CdS semiconductor nanoparticles involving Cd–citrate complex formation and controlled ion-exchange-assisted nucleation and growth, followed by separation, purification, drying, and powder processing to obtain phase-pure CdS nanostructures.

The aqueous Na_2_S solution was gradually added to the Cd–citrate precursor solution, forming a yellow suspension of CdS–SNC precipitate, as shown schematically in Figure 1. The stirring continued until all the nucleation and particle growth were completed. The resultant precipitate was then filtered off and thoroughly washed using deionised water and ethanol to remove any leftover precursor ions and impurities. Subsequently, the purified material was dried at 120–150 °C and then ground to obtain phase-pure CdS–SNC powder for further characterisation using photocatalytic, antibacterial and electrochemical studies.

### 2.3. Photocatalytic Studies

The photocatalytic performance of the synthesised CdS–SNCs was evaluated by monitoring the degradation of methylene blue (MB) as a model pollutant organic dye in aqueous solution by exposure to visible light. In a typical reaction, 100 mg of CdS particles were suspended in 100 mL of aqueous solution of methylene blue (10 ppm) under continuous stirring. Magnetic stirring was performed to obtain a uniformly distributed suspension of photocatalyst in the presence of organic dyes and prevent any precipitation of particles. Prior to illumination with visible light, the reaction mixture was subjected to magnetic stirring in the absence of light for 30 min. Exposure to visible light was achieved using a 36 W mercury lamp. A UV cut-off filter was placed between the light source and the reaction vessel to eliminate ultraviolet radiation, ensuring that only visible light (400–700 nm) was used for the photocatalytic experiments. The light intensity at the reaction surface was maintained at 100 mW cm^−2^. During the photocatalytic experiments, the reaction mixture was continuously stirred to ensure uniform dispersion of the photocatalyst and homogeneous irradiation. The reaction temperature was maintained close to room temperature throughout the experiment to minimise thermal effects associated with the mercury lamp, thereby ensuring that the observed degradation resulted predominantly from the photocatalytic process under visible-light irradiation. After every 30 min of irradiation, a specific amount of suspension was removed from the reactor and centrifuged to separate photocatalyst particles from the solution. The remaining concentration of methylene blue was measured by the characteristic absorption peak at 664 nm using UV–visible spectroscopy. When measuring absorbance, deionised water served as the baseline reference. The equation for photocatalytic degradation efficiency is as follows:

$$\mathrm{Degradation}\;\mathrm{efficiency}\left(\%\right)=\frac{C_{0}-C_{t}}{C_{0}}\times 100$$

where *C*_0_ and *C_t_* represent the initial and time-dependent concentrations (or absorbance values) of methylene blue, respectively [18,19,20].

The photocatalytic degradation kinetics were further analysed using a pseudo-first-order kinetic model based on the Langmuir–Hinshelwood mechanism. The apparent reaction rate constant (*k*) was determined from the slope of the linear plot of ln(*C*_0_/*C_t_*) versus irradiation time (*t*) according to the following Equation (1).

(1) $$\ln\left(\frac{C_{0}}{C_{t}}\right)=\mathrm{kt}$$

The calculated rate constant reflects the efficiency of photoinduced charge generation, charge separation, and surface catalytic activity of the CdS–SNCs under visible-light illumination [21,22,23].

### 2.4. Electrochemical Studies

The synthesised CdS–SNCs were characterised by cyclic voltammetry (CV) in a conventional three-electrode electrochemical configuration. The working electrode was a CdS-modified glassy carbon electrode (GCE), and the counter electrode and reference were made of platinum wire and saturated calomel electrode (SCE) respectively. Electrochemical measurements were performed at room temperature with a CHI660E electrochemical workstation (CH Instruments, Bee Cave, TX, USA). Before modification of the electrode, the surface of the glassy carbon electrode was sequentially polished with 0.3 and 0.05 µm alumina slurry to obtain a mirror-like finish. The polished electrode was then washed with deionised water, ultrasonically cleaned to remove excess alumina particles, and air-dried. Subsequently, the CdS nanocrystal was deposited on the clean GCE surface to obtain an electroactive interface suitable for electrochemical studies.

Electrochemical measurements were carried out primarily in aqueous solution of 0.1 M Na_2_SO_4_ electrolyte support medium. To investigate the effect of the electrolyte environment on the electrochemical behaviour of the CdS-modified electrode, additional measurements were performed in 0.1 M phosphate-buffered saline (PBS, pH 7.4) and 0.1 M KCl electrolytes. Deionised water was used to prepare all electrolyte solutions. The redox behaviour, charge storage capability, charge transfer kinetics, and electrochemical stability of the CdS-modified electrodes were evaluated by cyclic voltammetry measurements taken within an ideal potential window at various scan rates. To determine the application viability of synthesised CdS–SNCs in supercapacitor and energy storage applications, the electrochemical response was analysed.

### 2.5. Density Functional Theory Calculations

To study the electronic properties and charge transfer of the CdS semiconductor nanoclusters (CdS–SNCs), DFT calculations were conducted. The calculations were performed using the DMol3 module of BIOVIA Materials Studio software version 9.1. For the treatment of exchange–correlation energy, the GGA theory was applied by considering the Perdew–Burke–Ernzerhof Functional. The double numerical basis set with polarisation function (DNP) was used to represent the valence orbitals of the constituent atoms, whereas density functional semi-core pseudopotentials (DSPP) were used for the description of the core orbitals.

The CdS–SNCs were optimised without any consideration of symmetry until the convergence of the energy criteria of 1.0 × 10^−5^ Ha, force convergence criteria of 0.002 Ha Å^−1^, and atomic displacement of 0.005 Å was attained. SCF calculation of the energy was also carried out with the energy convergence criteria of 1.0 × 10^−6^ Ha. After optimisation, the FMO analysis and electronic structure calculation was performed.

The energies and spatial distribution of the HOMO and LUMO were evaluated by conducting frontier molecular orbital (FMO) analysis. The energy gap between HOMO and LUMO is called the HOMO-LUMO gap (ΔE) and determined using the relation

*ΔE* = *E_LUMO_* − *E_HOMO_*

Charge transfer characteristics of the CdS nanocluster were also investigated using charge density difference (CDD). Accumulation zones and depletion zones were depicted to pinpoint the polarisation zones accountable for charge separation. Optimised geometries, frontier molecular orbitals, and charge density distribution were studied to determine the correlation between the electronic features and the actual catalytic efficiency of the fabricated CdS–SNCs.

### 2.6. Antimicrobial Studies

The agar well diffusion method was used to evaluate the antimicrobial activity of the synthesised CdS–SNCs against selected bacterial and fungal strains. HiMedia, India Tryptone Soy Agar plate for bacterial culture and Sabouraud Dextrose Agar (SDA) for fungal culture were prepared under sterile conditions as mentioned above and poured into an aseptic Petri dish. For antibacterial studies, 100 µL inoculum was distributed homogeneously on the surface of bacterial growth agar plates by means of sterile glass spreader. Using a sterilised cork borer, wells of uniform diameter were created, followed by adding the CdS nanoparticle suspension in each well. Incubation of the inoculated plates was performed at 37 °C for 24 h followed by the evaluation of antibacterial activity through the measurement (mm) of zones of inhibition (ZI) generated around each well.

For the antifungal study, a stock solution of CdS nanoparticles (5 mg mL^−1^) was prepared by employing dimethyl sulfoxide (DMSO) as the dispersing medium [24,25,26]. The Sabouraud Dextrose Agar medium of pH 5.5 to 5.6 was autoclaved at 121 °C for 15 min followed by pouring into sterile Petri dishes of dimensions 100 mm × 15 mm. Once solidified, spores of the fungi were evenly inoculated in the medium, and holes of 8 mm diameter were drilled using sterile cork borer. Then, 100 µL of the prepared CdS nanosuspension was dispensed in each hole. Finally, the inoculated medium was left to incubate for 24 h at 29 °C.

The selected concentrations (5, 10, and 20 mg mL^−1^) were chosen to evaluate the concentration-dependent antibacterial response over low, intermediate, and high dosage levels.

Antimicrobial activity was assessed by measuring the inhibition-zone diameter around each well after incubation. The presence of definite zones showed the susceptibility of various microorganisms to CdS–SNCs, thus confirming their antimicrobial properties. This behaviour can be explained through several mechanisms, including the production of reactive oxygen species leading to oxidative stress, alteration in the integrity of the microbial cells’ membranes, and the interaction between nanoparticles and the microbial cell surface.

### 2.7. Material Characterisation

The structural characterisation and phase purity of synthesised CdS–SNCs were performed with powder X–ray diffraction (XRD) analysis using a Rigaku Ultima IV diffractometer (Rigaku Corporation, Tokyo, Japan) with Cu Kα radiation (λ = 1.5406 Å). Microstructure and surface morphology studies using FESEM, AFM and HRTEM energy-dispersive X-ray spectroscopy (EDAX) confirmed the elemental composition and chemical purity. Optical absorption measurements were performed with a Shimadzu UV-2600i UV–visible spectrophotometer. The electrochemical measurements were achieved by applying cyclic voltammetry (CV) in a standard three electrode cell, CdS coated glassy carbon electrode as working electrode, platinum wire as counter electrode and saturated calomel electrode (SCE) as a reference electrode using the CHI660E electrochemical workstation (CH Instruments, USA).

## 3. Results and Discussion

### 3.1. Structural, Morphological, and Compositional Characterisation

**Figure 2:**
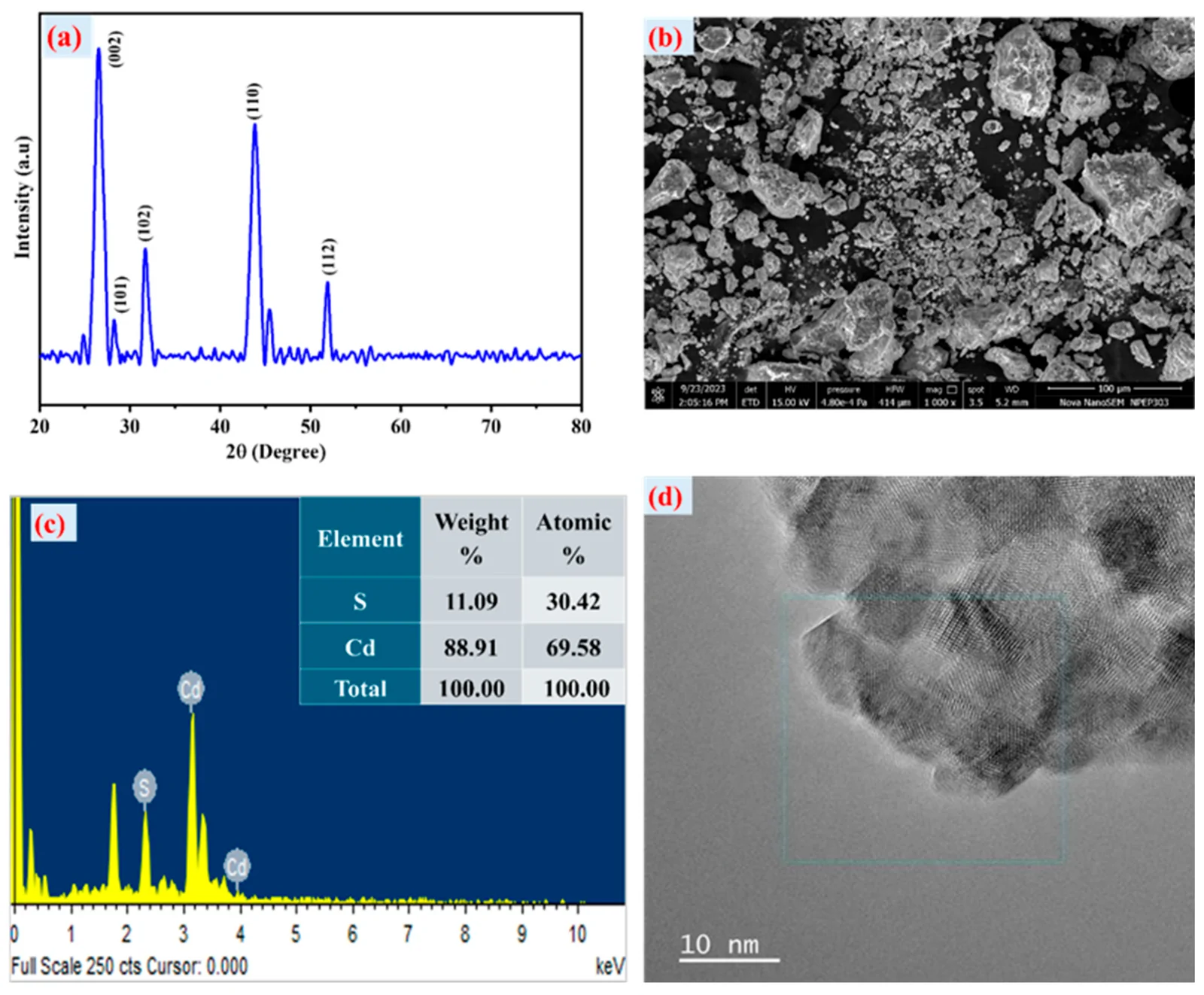
(**a**) XRD pattern of synthesised CdS–SNCs showing the hexagonal wurtzite phase, (**b**) FESEM image illustrating the agglomerated nanostructured morphology, (**c**) EDAX spectrum with elemental composition confirming the presence of Cd and S elements, and (**d**) HRTEM image displaying the nanocrystalline nature and well-defined lattice fringes of CdS–SNCs.

The crystal structure, phase purity, and crystallinity of the synthesised CdS–SNCs were investigated using powder X–ray diffraction (XRD), and the obtained diffraction pattern is shown in Figure 2a. The experimental diffraction profile was compared with the standard JCPDS reference pattern (Card No. 41-1049), confirming the formation of a phase-pure hexagonal wurtzite CdS structure [27]. All the observed diffraction peaks were successfully indexed to the characteristic crystallographic planes corresponding to the (002), (101), (102), (110), and (112) reflections of hexagonal CdS. No additional diffraction peaks associated with impurity phases such as CdO, Cd(OH)_2_, or other secondary compounds were detected, confirming the successful synthesis of single-phase CdS–SNCs with high chemical purity.

The crystallographic unit-cell parameters of the synthesised CdS semiconductor nanocrystals (CdS–SNCs) were determined from the indexed XRD pattern. The diffraction peaks were successfully indexed to the hexagonal wurtzite CdS crystal structure (JCPDS Card No. 41-1049, space group P6_3_mc). The calculated lattice parameters were a = b = 4.129 Å and c = 6.712 Å, yielding a c/a ratio of 1.625 and a unit-cell volume of 99.08 Å^3^. These crystallographic parameters are in good agreement with the characteristic hexagonal wurtzite structure of CdS and further confirm the successful formation of phase-pure CdS semiconductor nanocrystals. The calculated unit-cell parameters, together with the indexed diffraction peaks, validate the structural integrity and crystallinity of the synthesised material and are summarised in Table 1.

The observed peaks showed some level of broadening that implied that the synthesised compound had a nanocrystalline structure. By contrast, the observed relatively sharp and strong diffraction peaks suggested high crystallinity as well as an orderly, arranged lattice for the synthesised CdS–SNCs. This was estimated by using the formula for the Debye–Scherrer equation, as follows [28]:

(2) $$D=\frac{0.94\lambda }{\beta \cos\theta }$$

where λ = 1.5406 Å represents the wavelength of Cu Kα X-ray, β is the FWHM for the most intense diffraction peak, while θ represents the associated Bragg angle.

From the most intense diffraction peak, which occurred at 2θ = 26.52°, the mean crystallite size of the synthesised CdS–SNCs was obtained at about 6.2 nm [29].

The high crystallinity and phase purity confirmed by XRD are expected to contribute to improved structural stability and may facilitate charge transport. Similar correlations between crystallinity, charge transfer behaviour, and photocatalytic performance have been reported for semiconductor photocatalysts in the literature. However, direct evaluation of charge carrier separation was beyond the scope of the present study.

The XRD analysis confirms the successful formation of phase-pure hexagonal wurtzite CdS semiconductor nanocrystals without detectable secondary phases. The observed diffraction peak broadening is consistent with the nanocrystalline nature of the synthesised CdS–SNCs, yielding an average crystallite size of approximately 6.2 nm as estimated using the Debye–Scherrer equation. The peak broadening primarily reflects the small crystallite size of the material; however, the present XRD analysis alone does not permit a quantitative evaluation of lattice defects or microstrain. Therefore, no direct conclusions regarding defect density or lattice strain are drawn from the XRD results. The experimentally confirmed phase purity and nanocrystalline structure provide the structural basis for the multifunctional photocatalytic, electrochemical, and antibacterial performance discussed in the subsequent sections [30,31].

The morphology, microstructure, and elemental composition of the synthesised CdS–SNCs were examined using FESEM, HRTEM, and EDAX. The FESEM image in Figure 2b shows densely packed, interconnected structures with predominantly near-spherical features and relatively smooth surfaces. This aggregation may be associated with the high surface energy of the nanoparticles and van der Waals interactions between adjacent particles. Such morphology may influence charge transport and surface reactions and is therefore relevant to the photocatalytic and electrochemical applications discussed here [32].

The elemental constitution and the degree of chemical purity of the prepared CdS–SNCs were analysed by EDAX in connection with the FESEM technique, and its result is shown in Figure 2c. The absence of any signal related to impurity element apart from Cd and S in the EDAX spectra indicated the formation of pure phase CdS–SNCs. The ratio of Cd:S atoms was almost equal to that expected for the stoichiometry of CdS. An insignificant Cd-rich deviation in the elemental constitution might be due to the presence of sulphur vacancies at the surface and difference in X-ray ionisation cross-sections.

A deeper understanding of the nanoscale structure and crystallinity of the synthesised CdS–SNCs was acquired from the HRTEM investigation, as illustrated in Figure 2d. The HRTEM image reveals the formation of nanosized CdS particles with good crystallinity, as evidenced by the distinct lattice fringes. Although the particles appear nearly equiaxed, the HRTEM image alone does not permit a definitive assessment of particle shape. Therefore, morphology is described based on the observed nanocrystalline features rather than assigning a specific particle geometry. In addition, the HRTEM analysis showed well-defined lattice fringes, indicating the highly crystalline structure and ordered lattice structure of the synthesised CdS–SNCs [33,34]. The measured interplanar lattice spacing (d-spacing) of 0.336 nm corresponds to the (002) crystallographic plane of the hexagonal wurtzite CdS structure, which is in good agreement with the XRD analysis. The observed lattice fringes further confirm the high crystallinity and phase purity of the synthesised CdS nanocrystals.

The FESEM, HRTEM, and EDAX results indicate that phase-pure, crystalline CdS–SNCs have been successfully synthesised in a nanoscale interconnecting network structure. Such structural and morphological characteristics are expected to provide abundant active surface sites, enhanced light absorption, improved charge separation efficiency, and efficient ion/electron transport pathways, thereby contributing significantly towards the enhanced photocatalytic, antibacterial, and electrochemical performance of the synthesised CdS–SNCs.

### 3.2. Optical Properties and Electronic Structure Analysis

**Figure 3:**
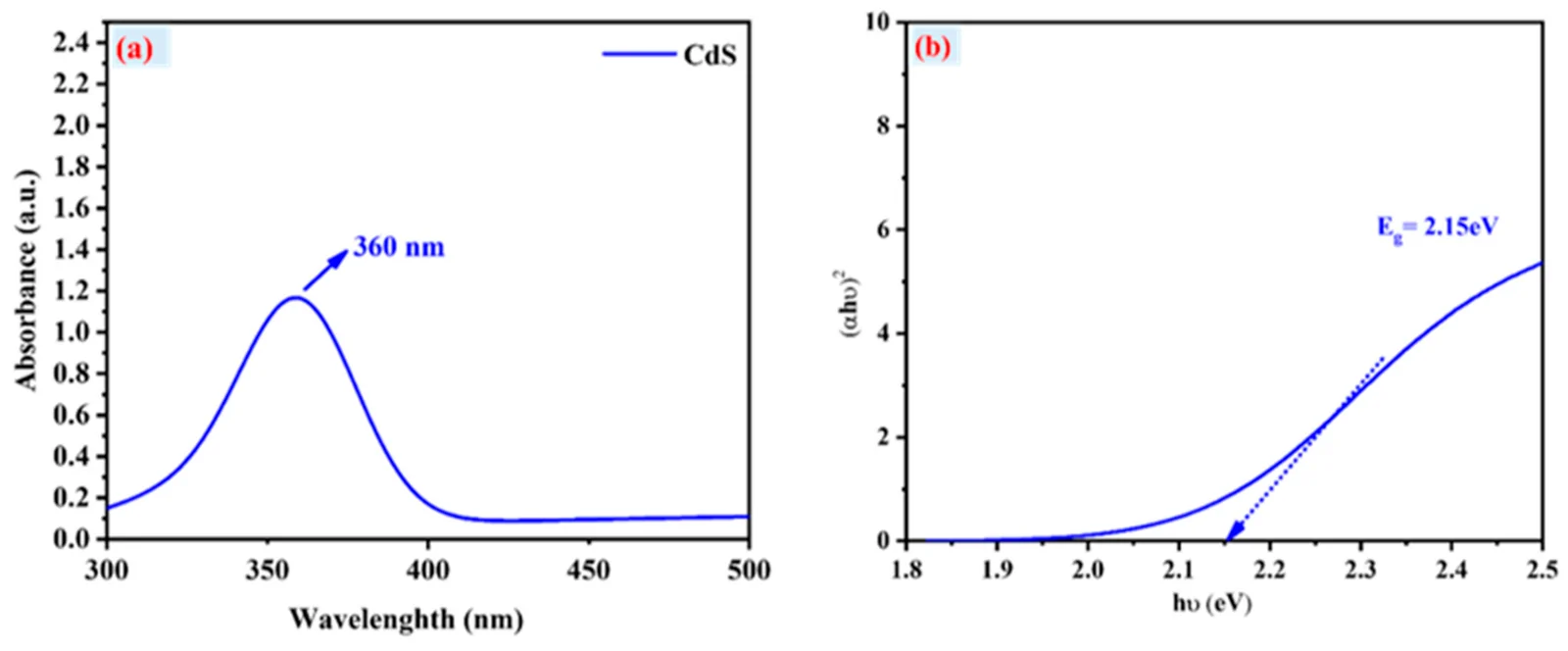
(**a**) UV–visible absorption spectra and (**b**) Tauc plots of the optical band gap of CdS–SNCs.

The optical properties of the synthesised CdS–SNCs were investigated using UV–visible absorption spectroscopy, and the corresponding spectra are presented in Figure 3a,b. The absorption spectrum shown in Figure 3a exhibits a pronounced excitonic absorption peak centred at approximately 360 nm, confirming the semiconducting nature and nanoscale dimensions of the synthesised CdS–SNCs.

Compared to bulk CdS, the absorption edge shows a noticeable shift towards the lower wavelength region, which can be attributed to the quantum confinement effect associated with the reduced particle size of the nanocrystals [35,36]. The observed absorption behaviour indicates effective visible-light absorption capability of the synthesised CdS–SNCs.

The energy of the optical band gap (*E_g_*) was obtained using the Tauc formula that applies to direct allowed electronic transition, as follows:

(*αhν*)^2^ = *A*(*hν* − *E_g_*) (3)

where *α* is the absorption coefficient, *hν* is the photon energy, *A* is material dependent constant and *E_g_* is the optical band gap energy.

Since CdS is a direct band gap semiconductor, a Tauc plot was obtained by plotting (*αhν*)^2^ against photon energy (*hν*), as presented in Figure 3b. The intercept on the photon energy axis gave the energy of the band gap.

The measured optical bandgap of 2.15 eV enables efficient visible-light absorption, which is favourable for photocatalytic activity. However, the overall photocatalytic performance is governed by multiple factors, including charge carrier separation, band-edge positions, surface characteristics, and charge transfer processes, in addition to the optical bandgap [37,38]. The difference in the band gap energy between nanostructured and bulk CdS could be because of nanosizing, reduced crystallite size, and quantum confinement that is evident in the synthesised nanostructures. In addition, high visible-light absorption capability and optimised band gap could favour improved efficiency in photocatalysis.

### 3.3. Visible-Light-Driven Photocatalytic Degradation Performance and Reaction Kinetics

**Figure 4:**
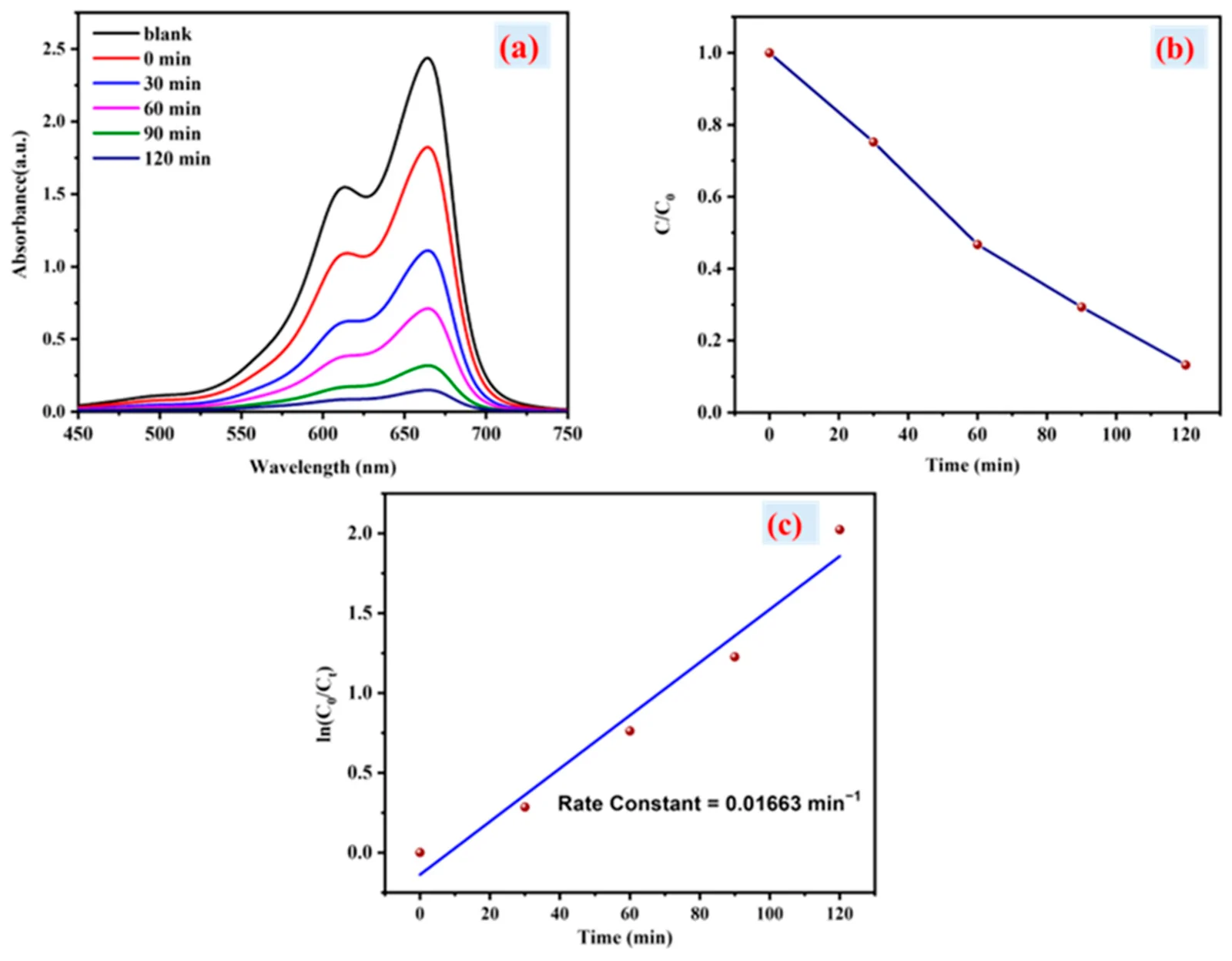
(**a**) UV–Vis absorption spectra showing photocatalytic degradation of methylene blue under visible-light irradiation at different time intervals, (**b**) variation in normalized concentration (*C*_t_/*C*_0_) with irradiation time, and (**c**) pseudo-first-order kinetic plot of ln(*C*_0_/*C*_t_) versus irradiation time for CdS–SNCs.

The prepared CdS–SNCs were evaluated for the photocatalytic degradation of methylene blue (MB) dye under visible-light irradiation. As shown in Figure 4a–c, the time-dependent UV–visible absorption spectra illustrate the changes observed during photocatalytic degradation. Prior to the irradiation, the reaction mixture was magnetically stirred in the dark for 30 min to achieve the adsorption–desorption equilibrium of photocatalyst surface and MB molecules. This suggests that MB molecules absorb on the surface of CdS–SNCs, and there is a slight decrease in initial absorbance intensity compared to the blank sample. The adsorption behaviour can be attributed to the high surface area and multiple active sites of nanocrystalline nature of the prepared CdS nanocrystals.

The pH of the methylene blue solution in the degradation study was kept constant at pH = 6.8 under ambient conditions. The steady decrease in the absorption intensity of methylene blue due to the increase in exposure time indicates that the degradation of the dye molecules is efficient when nanocrystals of cadmium sulphide are present. By contrast, the blank experiment conducted in the same visible light conditions without the use of a photocatalyst showed insignificant degradation, thereby proving that the dye degradation is mainly due to photocatalysis by the synthesised CdS–SNCs.

The photocatalytic degradation efficiency (η) was determined by the equation:

(4) $$\eta (\%)=\frac{C_{0}-C_{t}}{C_{0}}\times 100$$

where *C*_0_ and *C_t_* are the initial and time dependent concentrations of methylene blue, respectively.

The prepared CdS–SNCs had a photocatalytic degradation efficiency of about 89.93% after being exposed to visible light for 120 min, thus showing good photocatalytic activity. Figure 4b shows the plot of normalised dye concentration (C_t_/C_0_) versus irradiation time alongside a control experiment. The reduction in normalised concentration with increased irradiation time is an indication of the good degradation efficiency of methylene blue under visible-light irradiation in the presence of CdS–SNCs, while in the control experiment there was little or no change in the normalised concentration with time. The consistent reduction in normalised concentration indicates good and constant photocatalytic activity and reaction kinetics.

A limitation of the present photocatalytic investigation is the absence of dedicated ROS-scavenging and direct radical detection experiments. Such experiments, together with photoelectrochemical or time-resolved spectroscopic measurements, would provide stronger experimental evidence for identifying the dominant reactive species and clarifying the charge transfer mechanism. Future studies should therefore incorporate these techniques to establish the photocatalytic mechanism more conclusively.

To examine the kinetics of the photocatalytic reaction, the data obtained were analysed using pseudo first-order kinetic model. The kinetic equation can be expressed as:

(5) $$\ln\left(\frac{C_{0}}{C_{t}}\right)=\mathrm{kt}$$

where *k* is the apparent rate constant and *t* represents the irradiation time.

The linear relationship obtained from the plot of ln(*C*_0_/*C*_t_) versus irradiation time, shown in Figure 4c, confirms that the photocatalytic degradation process follows pseudo-first-order reaction kinetics. The linear fitting exhibited a high correlation coefficient (*R*^2^ = 0.992), further validating the applicability of the Langmuir–Hinshelwood kinetic model for the present photocatalytic system.

The observed photocatalytic performance may be associated with the nanocrystalline nature, visible-light absorption characteristics, and surface properties of the synthesised CdS semiconductor nanocrystals. However, the contributions of charge carrier separation and surface-active sites are discussed based on established literature rather than direct experimental confirmation in the present work. The reduced crystallite size and visible-light absorption characteristics are expected to facilitate photocatalytic activity, while the detailed charge transfer mechanism requires further investigation using advanced photoelectrochemical and spectroscopic techniques [39,40].

To provide a broader perspective on photocatalytic performance, the degradation efficiency of the synthesised CdS semiconductor nanocrystals was compared with representative CdS-based photocatalysts reported in the literature (Table 2). The present CdS nanocrystals achieved approximately 90% degradation of methylene blue within 120 min under visible-light irradiation, which is comparable to or better than several previously reported CdS-based photocatalysts under similar irradiation conditions. This comparison demonstrates the promising visible-light photocatalytic performance of the synthesised CdS nanocrystals for organic dye degradation.

#### 3.3.1. Photocatalytic Stability and Reusability

The practical applicability of photocatalysts can be assessed based on the operational stability, recyclability, and reproducibility. For instance, photocatalysts based on CdS usually experience photo corrosion and structural instability upon long exposure to visible-light irradiation. As a result, evaluation of photocatalytic durability and reusability becomes important in verifying the operational performance of CdS–SNCs.

The repeatability of the prepared CdS semiconductor nanocrystals was verified by conducting independent experiments on MB photocatalytic degradation under identical experimental conditions three times. Good repeatability and reliability were observed from the obtained results, indicating the excellent repeatability of the prepared photocatalyst [44,45]. On average, the degradation efficiency of MB was found to be 89.93% within 120 min of visible-light exposure, giving RSD = 1.24%.

**Figure 5:**
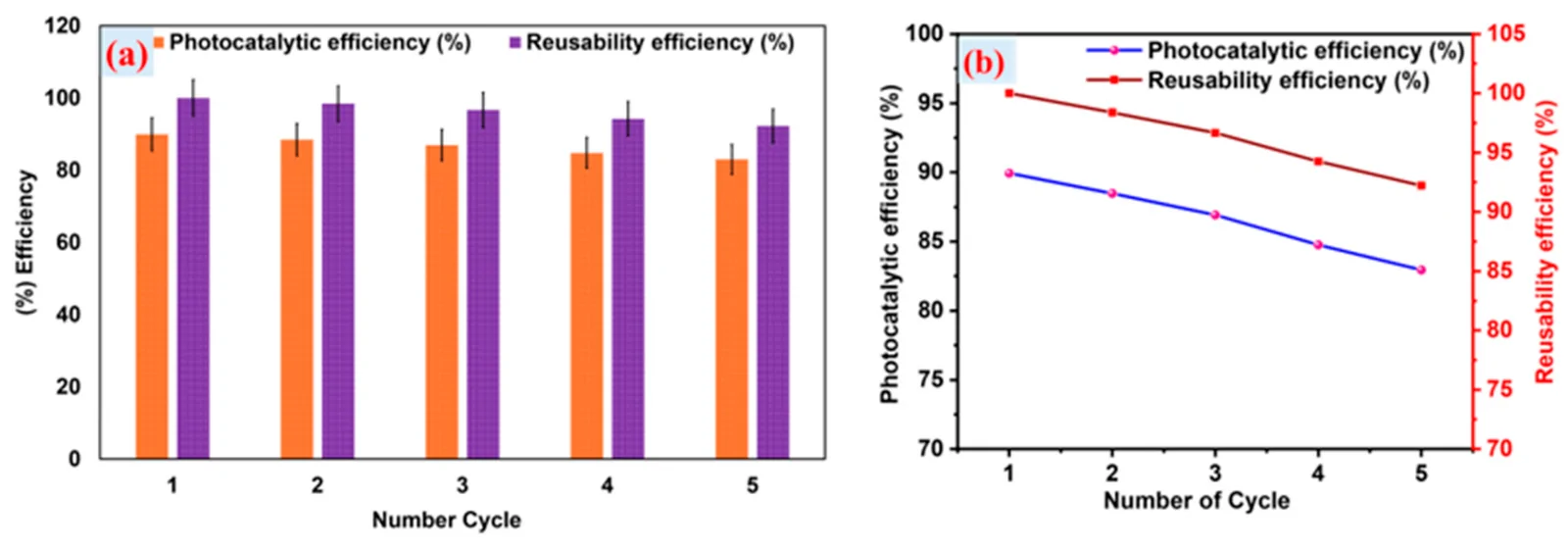
(**a**) Photocatalytic and reusability efficiencies (%) over five successive cycles. (**b**) Variation in efficiency with cycle number, demonstrating good stability and recyclability of the photocatalyst.

Moreover, the sustainability of the prepared CdS–SNCs in terms of their operational stability and recyclability was studied using five successive photocatalytic degradation cycles under the same conditions of visible light exposure. After each photocatalytic cycle, the CdS–SNCs were recovered by centrifugation, thoroughly washed several times with deionised water and ethanol to remove any adsorbed degradation products, and dried at 80 °C prior to reuse in the subsequent photocatalytic cycle. The corresponding recyclability performance and degradation retention efficiency are presented in Table 3 and Figure 5a,b respectively.

The synthesised CdS–SNCs retained approximately 92.22% of their initial photocatalytic activity even after five successive degradation cycles, demonstrating excellent structural stability and strong resistance towards photo corrosion under visible-light irradiation. The slight decrease in photocatalytic efficiency during subsequent cycles may be attributed to catalyst loss during recovery, partial blockage of surface-active sites, or the adsorption of reaction intermediates, formed through photocatalysis. Stability behaviour like this has been observed before in highly stable CdS visible light photocatalysts [46].

Significantly, post-cycling XRD results indicated no considerable shifts in diffraction peaks, crystallinity, and phases of the sample upon repeated photocatalytic processes, implying the maintenance of phase purity and structural integrity of the CdS–SNCs. The outstanding recyclability and structural stability can be explained by the high crystallinity, stable nanostructure morphology, efficient charge separation, and low electron–hole recombination properties of the prepared CdS–SNCs. The effective charge separation and prevention of photo corrosion have been reported to contribute significantly to the stability of the CdS-based photocatalysts. Overall, the outstanding reproducibility, high retention rate, and photocatalytic stability indicate that the environmentally friendly CdS–SNCs are highly stable in their photocatalytic operations.

Although the optical bandgap facilitates visible-light absorption, the photocatalytic efficiency of semiconductor nanocrystals is also influenced by the positions of the conduction and valence bands, charge carrier dynamics, and interfacial charge transfer processes. This study focuses on the structural, optical, photocatalytic, electrochemical, antibacterial, and theoretical characterisation of CdS semiconductor nanocrystals. Further investigations involving band-edge determination and photocurrent measurements would provide additional insight into the charge separation behaviour and photocatalytic mechanism and will be considered in future studies.

#### 3.3.2. Visible-Light Photocatalytic Mechanism

**Figure 6:**
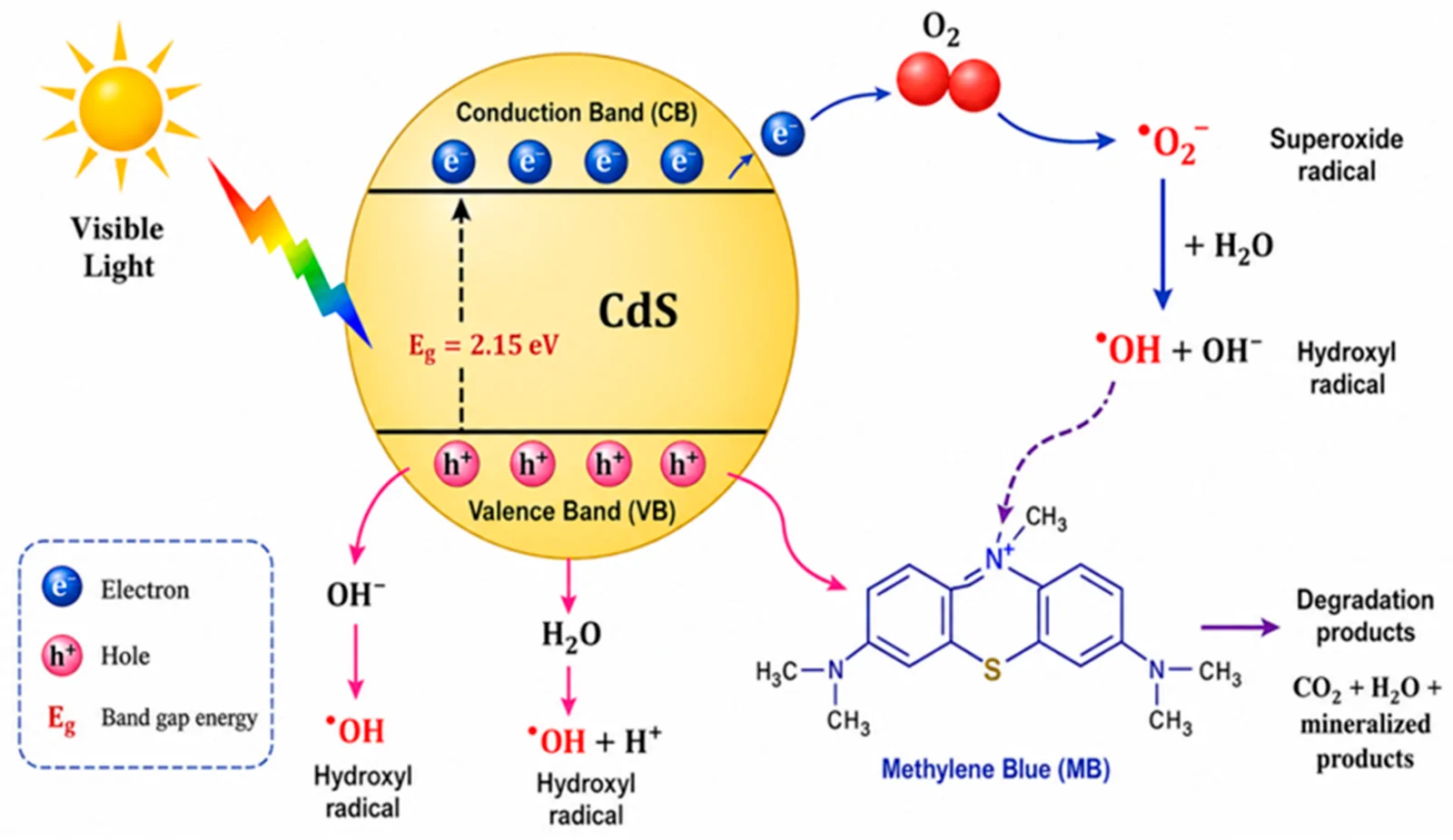
Schematic illustration of the visible-light photocatalytic mechanism of CdS–SNCs for methylene blue degradation via reactive oxygen species generation.

Figure 6 presents an illustration of the photocatalytic degradation process of methylene blue (MB) by CdS–SNCs under visible-light irradiation. Based on the optical properties and photocatalytic performance of the synthesised CdS semiconductor nanocrystals, a plausible photocatalytic mechanism is proposed. Under visible-light irradiation, photogenerated electrons are excited from the valence band to the conduction band, leaving corresponding holes in the valence band. These charge carriers may participate in surface redox reactions, leading to the formation of reactive oxygen species (ROS), including superoxide radicals (O_2_^−^) and hydroxyl radicals (•OH), which are widely recognised as the primary oxidising species responsible for the degradation of organic pollutants. However, since ROS scavenger or direct ROS detection experiments were not performed in the present study, the involvement of these reactive species should be considered a literature-supported interpretation rather than direct experimental evidence. Similar photocatalytic pathways have been widely reported for CdS-based semiconductor photocatalysts.

The small band gap and high efficiency of visible-light absorption led to the higher photocatalytic activity of CdS–SNCs. Under visible-light irradiation, photons with the energy equal to or more than the band gap energy generated electron–hole pairs (h^+^VB/e^−^CB) by exciting electrons from the valence band (VB) to the conduction band (CB) [47].

The photogenerated electrons in the conduction band subsequently react with dissolved oxygen molecules adsorbed on the CdS surface to produce superoxide radicals:

$$O_{2}+e^{-}\to \cdot O_{2}^{-}$$

The generated superoxide radicals further interact with water molecules to form highly reactive hydroxyl radicals:

$$\cdot O_{2}^{-}+H_{2}O\to \cdot \mathrm{OH}+OH^{-}$$

Simultaneously, the photogenerated holes present in the valence band oxidise hydroxide ions and water molecules, producing additional hydroxyl radicals through the following reactions:

*h*^+^ + *OH*^−^ → ⋅*OH*

*h*^+^ + *H*_2_*O* → ⋅*OH* + *H*^+^

Based on the optical properties and photocatalytic degradation results, a plausible visible-light photocatalytic pathway is proposed. Upon irradiation with visible light, CdS nanocrystals can generate photogenerated electron–hole pairs that may participate in surface redox reactions. The photogenerated electrons may contribute to the reduction of adsorbed oxygen to reactive oxygen species such as superoxide radicals (•O_2_^−^), while photogenerated holes may participate in oxidation reactions that can contribute to the formation of hydroxyl radicals (⋅*OH*). These processes can subsequently contribute to the degradation of organic dye molecules. However, the present study did not include radical trapping/scavenging experiments or direct ROS detection. Therefore, the involvement of $\cdot O_{2}^{-}$, and •OH is proposed as a plausible mechanism based on the established photocatalytic behaviour of CdS and relevant literature, rather than being considered direct experimental evidence from the present study [48].

The generated reactive oxygen species (ROS), mainly hydroxyl radicals (⋅*OH*) and superoxide radicals ($\cdot O_{2}^{-}$), possess strong oxidative capability and actively attack the adsorbed methylene blue molecules, leading to their degradation into smaller intermediate species and, eventually, mineralised products:

$$\mathrm{MB}+\cdot \mathrm{OH}/\cdot O_{2}^{-}\to CO_{2}+H_{2}O+\mathrm{mineralized}\;\mathrm{products}$$

Therefore, the enhanced photocatalytic degradation performance of the synthesised CdS–SNCs originates from the synergistic effect of efficient visible-light absorption, effective charge carrier separation, and continuous generation of reactive oxygen species under visible-light irradiation [49,50].

### 3.4. Electrochemical Energy Storage Properties

The electrochemical energy storage properties of the synthesised CdS–SNCs were systematically investigated using cyclic voltammetry (CV), galvanostatic charge–discharge (GCD), and electrochemical impedance spectroscopy (EIS) measurements in a conventional three-electrode configuration. The CdS-coated glassy carbon electrode (GCE) was employed as the working electrode, while platinum wire and a saturated calomel electrode (SCE) served as the counter and reference electrodes, respectively. All electrochemical measurements were performed in 1 M Na_2_SO_4_ aqueous electrolyte at room temperature.

The electrochemical measurements were conducted using a three-electrode configuration, with the prepared CdS electrode serving as the working electrode, together with the reference and counter electrodes described in the experimental procedure. The electrochemical measurements and calculated specific capacitance values should therefore be interpreted within this three-electrode configuration. Two-electrode full-cell/device measurements were not performed in the present study; consequently, the reported results are considered to represent the electrochemical response of the prepared CdS material rather than practical full-device performance.

#### 3.4.1. Cyclic Voltammetry (CV)

**Figure 7:**
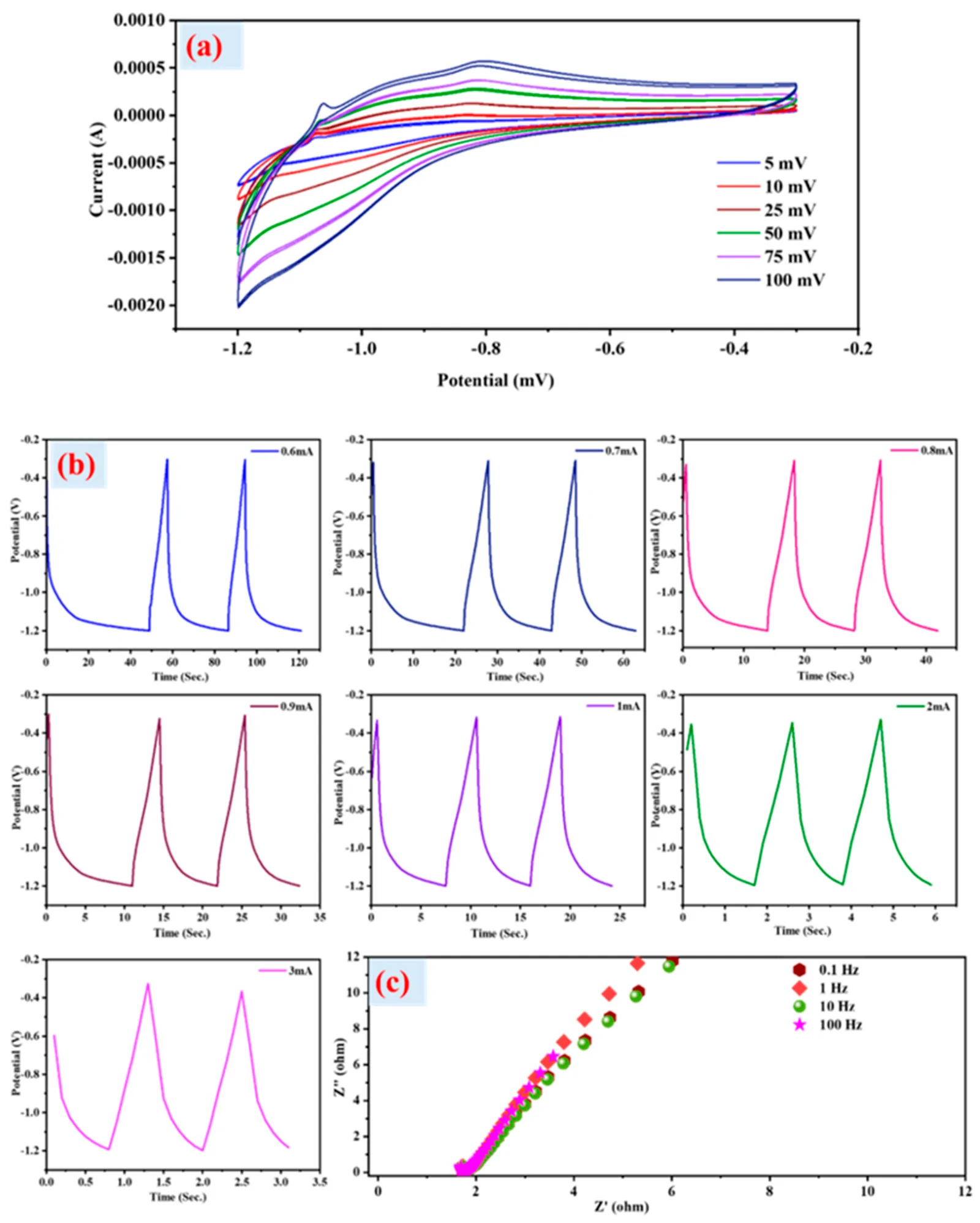
(**a**) CV curves at different scan rates, (**b**) GCD profiles at various current densities, and (**c**) electrochemical performance analysis of the synthesised electrode material for supercapacitor applications.

Capacitance of the CdS–SNC electrode was measured through cyclic voltammetry at different scan rates ranging from 5 to 100 mV s^−1^ within the potential range of −1.2 to −0.2 V. The CV curves for the different scan rates are shown in Figure 7a–c. The voltammogram was almost symmetric and square-shaped, suggesting good capacitive properties, fast charge transfer and good electrochemical reversibility of the CdS electrode.

Specific capacitance (Cs) was measured using the equation:

(6) $$C_{s}=\frac{1}{\mathrm{mv}(V_{c}-V_{a})}\int_{V_{a}}^{V_{c}}I(V)\mathrm{dV}$$

where *I*(*V*) stands for the current response, *v* stands for the scan rate (V s^−1^), *m* is the mass of the electroactive CdS material deposited onto the electrode, and (*V_c_* − *V_a_*) represents the potential window of application.

The obtained CdS–SNCs exhibited a specific capacitance value of 17.75 F g^−1^ at a scan rate of 5 mV s^−1^. The reduction in the specific capacitance values with an increase in the scan rate may result from insufficient ion diffusion to the electroactive sites in the case of fast polarisation processes [51,52]. The excellent retention of capacitive behaviour at higher scan rates, as observed in the CV profiles (Figure 7a) and summarised in Table 4, confirms efficient ion-diffusion kinetics, rapid charge transfer characteristics, and good electrochemical stability of the synthesised CdS–SNCs during repeated electrochemical operation.

#### 3.4.2. Galvanostatic Charge–Discharge (GCD)

The charge storage capacity and electrochemical reversibility of the CdS electrode was additionally assessed using galvanostatic charge–discharge (GCD) tests at current densities between 0.6 and 3 mA cm^−2^, illustrated in Figure 7b. To make the comparative analysis easier, all GCD data for various current densities was plotted on a single graph. The charge/discharge curves are almost perfectly symmetrical and triangle-shaped, meaning that they represent ideal capacitors with high reversibility and extremely high coulombic efficiency. The linear shape of the GCD curves suggests low internal resistance and efficient transport of charges along the electrode/electrolyte boundary [53,54]. Moreover, the long discharge time at low current density indicates improved charge storage capacity of the CdS nanostructured electrode.

#### 3.4.3. Electrochemical Impedance Spectroscopy (EIS)

The charge transfer characteristics and ion diffusion properties at the CdS electrode/electrolyte interface were studied using electrochemical impedance spectroscopy. The Nyquist plot obtained is shown in Figure 7c.

The impedance plot exhibits a small intercept in the high-frequency region, corresponding to the solution resistance (R_s_). The impedance response in the low-frequency region reflects the combined contributions of the electrochemical processes associated with the electrolyte, active material, and current collector.

The indistinct semicircle of the high-frequency region indicates a low charge transfer resistance (*R_ct_*), which means that rapid electron transfer can occur at the electrode–electrolyte interface [55,56]. In the low-frequency region, the approximately vertical line observed in the Nyquist plot indicates predominantly capacitive behaviour, suggesting effective charge storage and favourable ion-transport characteristics.

Equivalent circuit analysis was performed by fitting the impedance data to a modified Randles circuit model comprising (*R_s_*), (*R_ct_*), CPE and W. Analysis of the equivalent-circuit parameters confirms low interfacial resistance and favourable ion-diffusion behaviour, hence the excellent electrochemical properties exhibited by the CdS semiconductor nanocrystals. The low value of (*R_ct_*) further suggests rapid Faradaic charge transfer reaction [57,58].

The superior electrochemical properties of the CdS–SNCs could be due to their small size, large surface area, and nanostructured nature that enable fast electron transfer and increased electrolyte access. It is evident from the results obtained from CV, GCD, and EIS analysis that the material shows excellent charge storage property, low interfacial resistance, and favourable electrochemical kinetics, indicating the suitability of CdS–SNCs for electrochemical energy storage applications [59].

It should be noted that the specific capacitance of 17.75 F g^−1^ obtained in the present study primarily reflects the intrinsic electrochemical behaviour of pristine CdS semiconductor nanocrystals. This value is lower than those reported for CdS-based composites, heterostructures, or carbon-supported electrodes, where conductive matrices and synergistic interfacial effects significantly improve electrical conductivity, charge transport, and electrolyte accessibility. In contrast, the present work intentionally evaluates pristine CdS nanocrystals to establish their inherent electrochemical characteristics. Despite the moderate specific capacitance, the material exhibited good cycling stability, low charge transfer resistance, and favourable electrochemical kinetics, demonstrating its potential as a model semiconductor electrode for further material optimisation.

The electrochemical behaviour observed in the present study is consistent with the structural characteristics, phase purity, and nanocrystalline nature of the synthesised CdS semiconductor nanocrystals. Conversely, the detailed charge transfer mechanism and the contribution of charge carrier dynamics have not been directly investigated in the present work. Further studies employing advanced spectroscopic and photoelectrochemical techniques, such as photocurrent and impedance analyses under illumination, would provide a more comprehensive understanding of the electrochemical charge transfer process.

#### 3.4.4. Cycling Stability and Electrochemical Durability

The cycling stability and electrochemical stability of the developed CdS nanocrystals electrode material were systematically tested by performing multiple CV scans in 1 M Na_2_SO_4_ aqueous electrolyte at the scanning rate of 50 mV s^−1^. Cycling stability is regarded as one of the important criteria to test the suitability of electrode materials for their use in electrochemical energy storage devices like supercapacitors [60,61].

**Figure 8:**
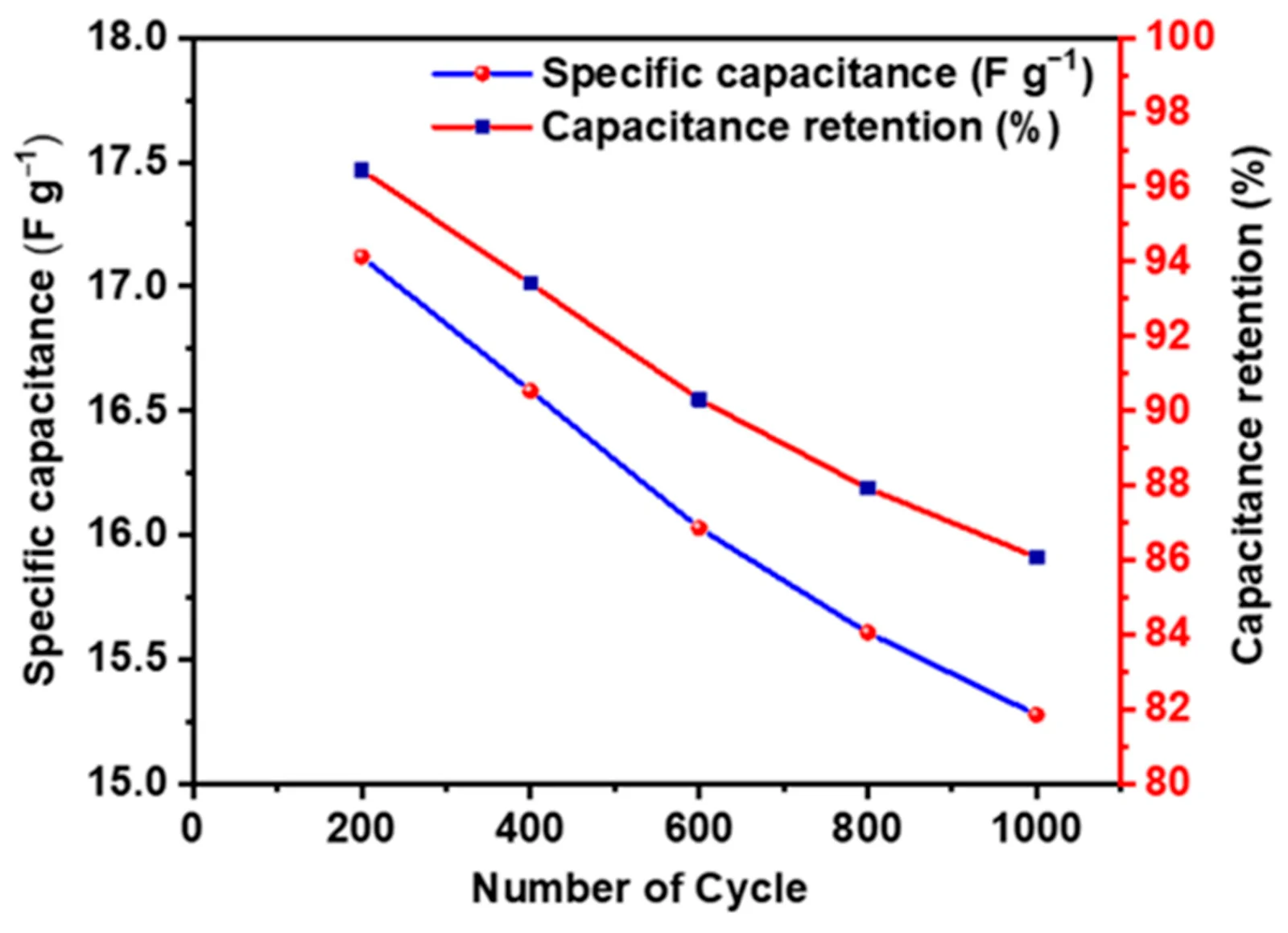
Electrochemical cycling stability and capacitance retention behaviour CdS–SNC electrode over 1000 charge–discharge cycles in 1 M Na_2_SO_4_ electrolyte.

The electrochemical cycling behaviour of the CdS electrode was analysed by performing 1000 electrochemical cycles in succession, and the specific capacitance retention data are listed in Table 5 and Figure 8. The as-synthesised CdS nanocrystal semiconductor electrode showed very good electrochemical stability, as there was just a slow decline in specific capacitance upon long-term cycling.

The CdS semiconductor nanocrystals showed about 86.08% retention of its specific capacitance after 1000 cycles, indicating good electrochemical reversibility and stability of the charge storage mechanism. This is because, due to repetitive cycling, there might have been slight surface reconstruction and blocking of electroactive sites, as well as some deformation in structure resulting from continuous ion insertion/extraction process [62].

There are several reasons for the stability of the CdS electrode during cycling, which include high crystalline structure, phase stability, interconnected nanostructures, ion diffusion efficiency, low charge transfer resistance, and enhanced access to the electrolyte ions by the CdS nanostructure. Furthermore, the low resistance between the electrolyte and electrode materials and efficient transport of electrons observed from EIS measurement play a major role in maintaining capacitive stability during repetitive electrochemical cycling. Other similar behaviours were seen in stable semiconductor supercapacitor electrode materials that exhibit good, interconnected nanostructures and efficient electrolyte ion transport [63,64].

The sustained electrochemical activity even after long-term cycling also proves the stability of the CdS electrode/electrolyte interface and durability of the fabricated nanocrystals during repeated electrochemical testing. This indicates, from the above results, that the environmentally friendly synthesised CdS–SNCs exhibit good electrochemical durability and stability, making them promising candidates for future electrochemical energy storage devices.

### 3.5. DFT-Based Electronic Structure and Structure–Property Analysis

**Figure 9:**
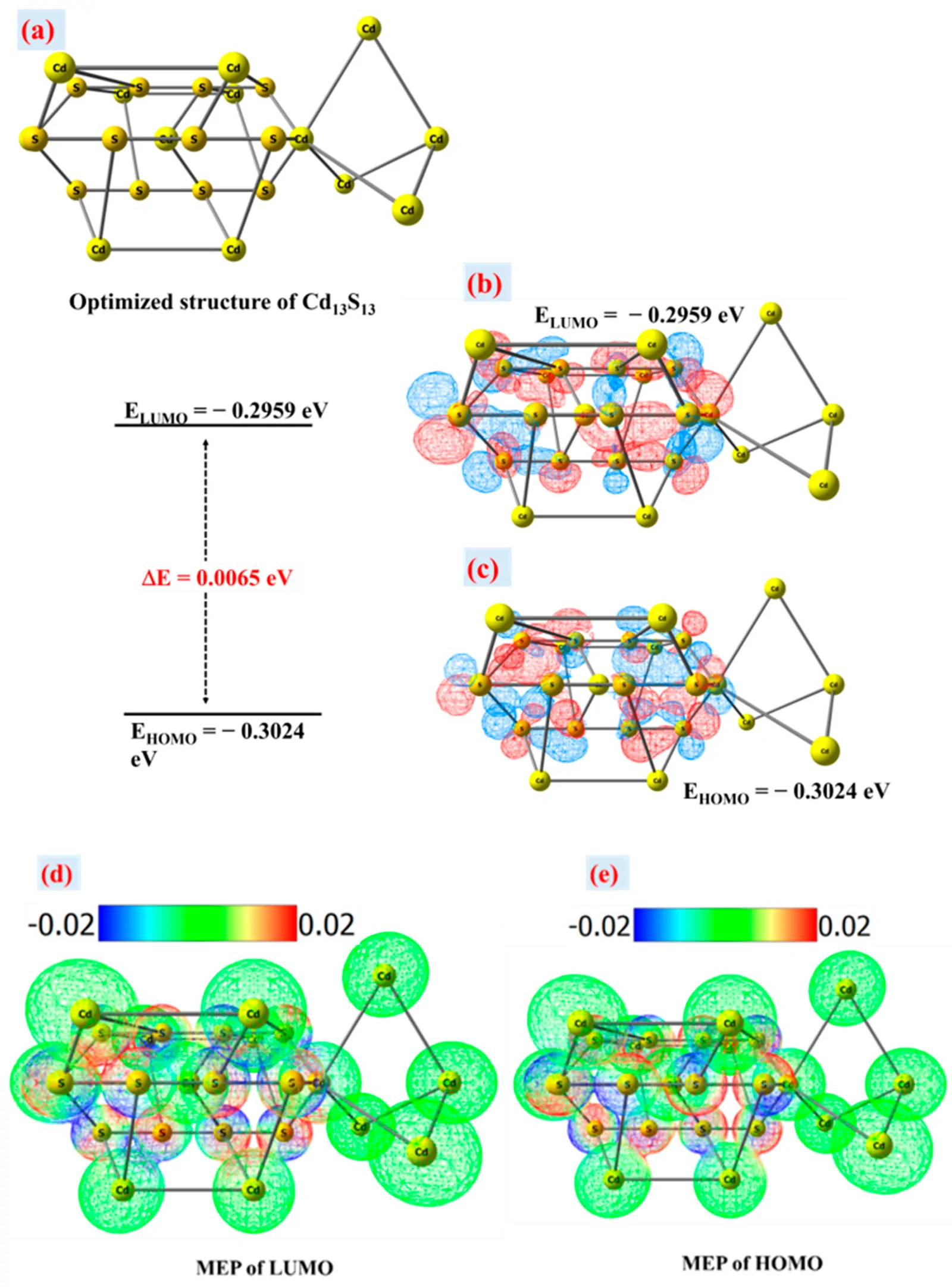
DFT analysis of CdS semiconductor nanocluster showing (**a**) optimised atomic structure, (**b**) LUMO distribution, and (**c**) HOMO distribution with narrow HOMO–LUMO energy gap (Δ*E*= 0.0065 eV) facilitating efficient charge transfer. (**d**,**e**). Charge density difference (CDD) maps of CdS semiconductor nanocluster showing electron accumulation and depletion regions associated with charge redistribution and polarisation within the Cd–S framework.

Density functional theory (DFT) calculations were performed to gain further insight into the electronic structure, charge transfer, and multifunctionality of the synthesised CdS–SNCs. The optimised atomic structures and their corresponding electronic properties for the CdS nanoclusters are shown in Figure 9a. The geometry optimisation established the stability of the Cd-S bonding structure by confirming energetically favourable coordination of cadmium with sulphur atoms that leads to efficient delocalisation of electrons.

Such spatial separation of the frontier orbitals indicates a possible directionality of electronic redistribution within the finite cluster model. It provides qualitative information about charge distribution, but it does not by itself establish suppressed electron–hole recombination in the experimental semiconductor.

Frontier molecular orbital (FMO) analysis indicated the spatial separation between the highest occupied molecular orbital (HOMO) and lowest unoccupied molecular orbital (LUMO) as illustrated in Figure 9b,c. The HOMO was found to be primarily distributed at the sulphur atom while the LUMO was mostly localised at the cadmium atom [65,66]. Such localisation of FMO enhances directional charge transfer of electrons from the sulphur to the cadmium site, hence inhibiting electron–hole recombination.

It should be noted that the HOMO–LUMO energy gap obtained from the finite CdS nanocluster model represents the electronic structure of the molecular cluster and should not be directly compared with the experimentally determined optical bandgap obtained from UV–visible spectroscopy. The experimental optical bandgap (2.15 eV) corresponds to electronic transitions in the semiconductor nanocrystals, whereas the calculated HOMO–LUMO gap primarily provides qualitative insight into the electronic structure, charge distribution, and charge transfer characteristics of the model system. Similar distinctions between theoretical molecular orbital gaps and experimental optical bandgaps have been reported in the literature [67].

The DFT calculations provide qualitative insight into the electronic structure and charge distribution of the CdS nanocluster. The relationship between these electronic characteristics and the experimentally observed multifunctional performance is discussed with support from previous studies rather than being interpreted as direct experimental verification. Furthermore, the calculated HOMO–LUMO energy gap represents the electronic characteristics of the finite cluster model and should not be directly compared with the experimentally determined optical bandgap of the CdS semiconductor nanocrystals.

The theoretical electronic energy levels showed that the energy level of LUMO (*E_LUMO_* = −0.2959 eV) is more favourable for the reduction of dissolved oxygen molecules to superoxide anions ($\cdot O_{2}^{-}$), while the energy level of HOMO (*E_HOMO_* = −0.3024 eV) is favourable for oxidation reactions of surface hydroxyl groups and water molecules. The energy gap (Δ*E*) between HOMO and LUMO was determined as follows:

Δ*E* = *E_LUMO_* − *E_HOMO_*

The calculated HOMO–LUMO energy gap of approximately 0.0065 eV represents the electronic structure of the finite CdS nanocluster model used in the DFT calculation. This value should not be directly compared with or interpreted as the experimental optical bandgap of the CdS semiconductor. The experimentally determined optical bandgap of the synthesised CdS nanocrystals was approximately 2.15 eV based on the UV–Vis absorption analysis. The difference between these quantities arises from their fundamentally different physical descriptions: the experimental value represents the optical transition characteristics of the semiconductor material, whereas the calculated HOMO–LUMO gap describes the frontier-orbital separation of the finite molecular/cluster model. Therefore, the DFT-derived HOMO–LUMO gap is considered here as a qualitative descriptor of the electronic structure, electronic softness, and charge redistribution of the model rather than as a prediction of the experimental semiconductor bandgap.

Additional information regarding the electronic charge transfer within the CdS nanoclusters was gained from charge density difference (CDD) analysis shown in Figure 9d,e. CDD analysis demonstrated a high depletion of electrons around cadmium atoms and electron accumulation around sulphur atoms, indicating strong polarisation of the bonds between Cd and S atoms due to the differences in their electronegativities. Such charge polarisation can create local internal electric fields that promote charge separation and prevent electron–hole recombination.

Electron-deficient sites of cadmium atoms serve as active centres for oxygen adsorption and activation and thus accelerate the generation of superoxide radicals ($\cdot O_{2}^{-}$), while electron-rich sites of sulphur atoms promote oxidation reactions mediated by holes, resulting in hydroxyl radical (⋅OH) formation. This bifunctional behaviour of active centres is highly important in enhancing the photocatalytic degradation efficacy and redox activity of surfaces [68]. The DFT results on the electronic properties of synthesised nanoparticles are in good accordance with the experimentally obtained photocatalytic, electrochemical, and antimicrobial activities of CdS–SNCs. Efficient charge separation and ROS production calculated via DFT are consistent with the experimentally measured 89.93% methylene blue degradation rate under visible-light irradiation.

Moreover, the increased electronic delocalisation and continuous charge transport pathways predicted from DFT calculations are supported by low charge transfer resistance and excellent electrochemical performance achieved through the analysis of cyclic voltammetry and electrochemical impedance spectroscopy studies. The specific capacitance value of 17.75 F g^−1^ measured experimentally is another confirmation of the efficient electrochemical charge storage performance of the synthesised CdS–SNCs.

The antimicrobial activity shown against *Staphylococcus aureus*, *Pseudomonas aeruginosa*, and *Candida albicans* can also be explained in terms of electronic structure derived from DFT calculations. The preference for LUMO electrons’ localisation near the cadmium atoms helps the nanoparticles interact with electron-rich functional groups of the microbial cell membranes, which, together with ROS formation under physiological conditions, results in oxidative stress and membrane disruption, leading to microbial cell inactivation [69,70].

In conclusion, high concordance between experimental results and DFT calculations provides evidence that there is a structure–property–performance relationship established for the synthesised CdS–SNCs. This integrated approach proves that multifunctional properties of CdS nanostructures are determined by their electronic structure, charge transport ability, and ROS production, thus making them highly promising materials for various photocatalytic, antimicrobial, and electrochemical energy storage applications.

### 3.6. Antimicrobial Activity

To evaluate the antimicrobial efficiency of the synthesised CdS–SNCs, experiments were performed using the agar well diffusion method for Gram-positive *Staphylococcus aureus*, Gram-negative *Pseudomonas aeruginosa* and fungal *Candida albicans strains*. The results proved that the synthesised CdS–SNCs, possess concentration-dependent antimicrobial efficiency.

The antibacterial experiments were conducted under the experimental conditions described in the methodology section. The tested CdS concentrations, microbial inoculum, incubation conditions, and inhibition-zone measurement procedure were clarified to improve experimental reproducibility. The inhibition-zone measurements are reported as experimentally obtained, and the corresponding figure captions have been revised to specify the measurement conditions and replicate information, where available.

#### 3.6.1. Antibacterial Activity

The antibacterial activity of CdS–SNCs against the Gram-positive bacterium *Staphylococcus aureus* and Gram-negative bacterium *Pseudomonas aeruginosa* was investigated using the agar well diffusion method. The wells were prepared with three different concentrations of CdS–SNC suspension (5, 10, and 20 mg mL^−1^), which were used to prepare the wells, while Ciprofloxacin (1 mg mL^−1^) was used as a positive control and DMSO as a negative control. Bacterial suspensions were inoculated on the obtained agar plates before leaving to incubate at 37 °C overnight for ZOI observation.

The inhibition zone diameter increased consistently with increasing CdS–SNC concentration, demonstrating a clear concentration-dependent antibacterial response against both Gram-positive and Gram-negative bacteria. Although only three representative concentrations were evaluated in the present study, the observed trend provides evidence of dose-dependent antimicrobial activity. A more detailed concentration–response study involving additional concentration levels will be considered in future work to further quantify the antibacterial efficacy.

**Figure 10:**
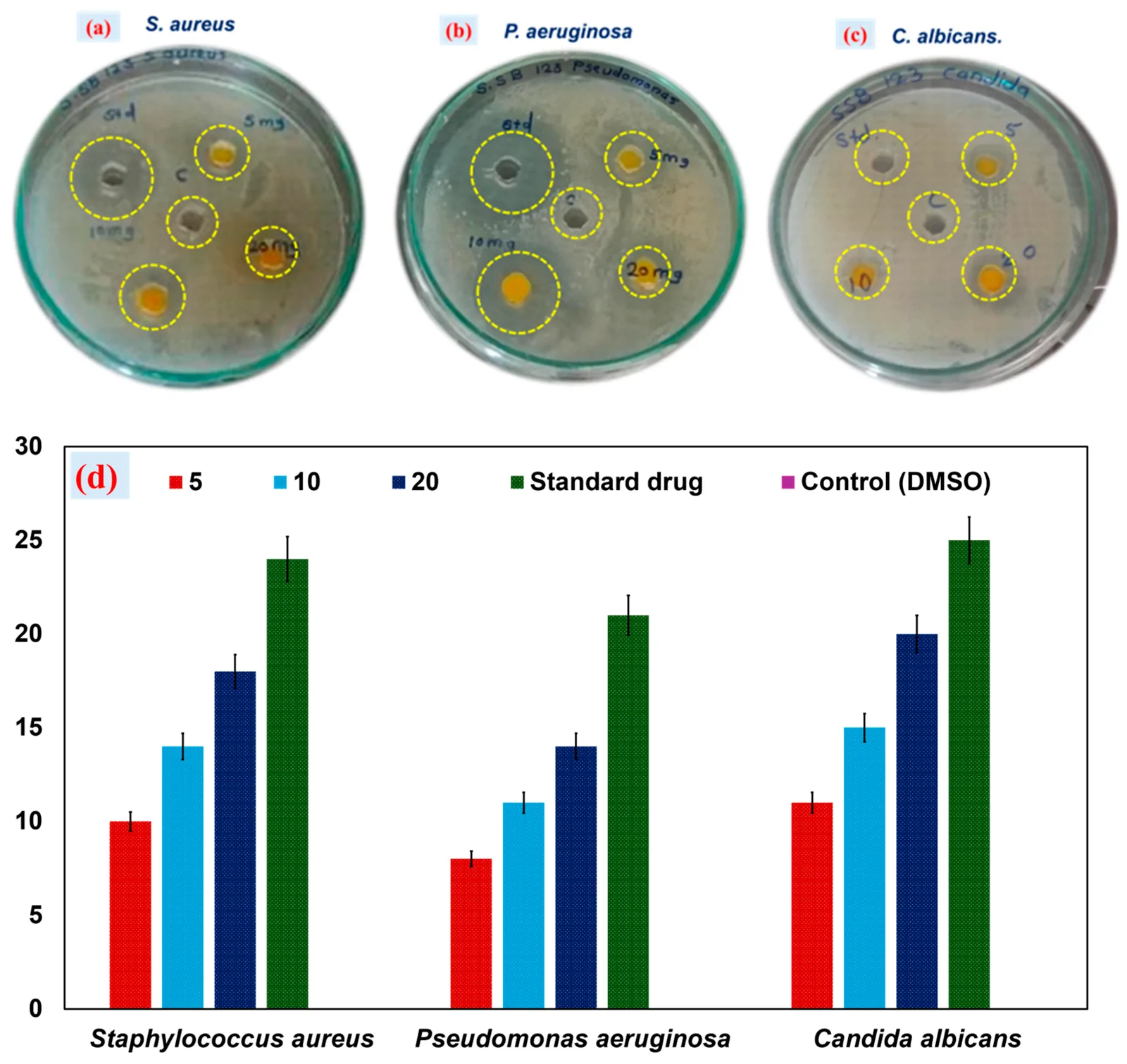
(**a**–**c**). Antimicrobial activity of CdS semiconductor nanocrystals against *Staphylococcus aureus*, *Pseudomonas aeruginosa*, and *Candida albicans* at different concentrations, showing concentration-dependent zones of inhibition. (**d**). Comparative antibacterial and antifungal activities of CdS semiconductor nanocrystals against *Staphylococcus aureus*, *Pseudomonas aeruginosa*, and *Candida albicans* at different concentrations, showing concentration-dependent enhancement in zone of inhibition (ZOI) relative to standard drug and control.

As seen in Table 6 and Figure 10a,b, the prepared CdS nanocrystals showed excellent concentration-dependent antibacterial activity towards the tested bacteria. The maximum efficacy was achieved at a concentration of 20 mg mL^−1^ where the zones of inhibition were found to be 18 ± 1 mm for *Staphylococcus aureus* and 14 ± 1 mm for *Pseudomonas aeruginosa*. In general, the Gram-positive bacteria are more sensitive due to their simple cell wall structure, while the Gram-negative bacteria have an additional layer of outer membrane which limits the diffusion of nanoparticles and reactive oxygen species [71,72].

#### 3.6.2. Antifungal Activity

The antifungal activity of the synthesised CdS–SNCs was investigated using *Candida albicans* via Sabouraud dextrose agar (SDA) medium. CdS–SNCs were dispersed in dimethyl sulfoxide (DMSO) at concentrations of 5, 10, and 20 mg mL^−1^, while clotrimazole (1 mg mL^−1^) was used as the standard antifungal compound. Inoculated plates were incubated at 29 °C for 24 h, and antifungal activity was measured by determining the inhibition zones as shown in Figure 10c.

It was found that synthesised CdS–SNCs showed outstanding antifungal activity with increasing inhibition zones with increasing concentration. Antifungal activity was highly effective for 20 mg mL^−1^, showing that there is strong inhibition of fungal growth. Synthesised CdS–SNCs showed similar antifungal activity as the standard clotrimazole drug, which demonstrates that synthesised CdS–SNCs have good antifungal activity [73,74]. The prepared CdS–SNCs demonstrated a notable concentration-dependent antimicrobial activity on the Gram-positive bacterium *Staphylococcus aureus*, the Gram-negative bacterium *Pseudomonas aeruginosa*, and the fungus *Candida albicans*, as depicted in Figure 10d. Increased antimicrobial efficiency at higher concentrations of CdS is due to an increased interaction between the surface of the nanoparticles and microbial cells, leading to the formation of reactive oxygen species (ROS) that eventually cause membrane damage and oxidative stress. As compared to other microbes used in this study, *Candida albicans* and *Staphylococcus aureus* displayed high sensitivity towards CdS nanoparticles.

The antibacterial activity of the synthesised CdS nanocrystals was evaluated using the inhibition-zone method. The observed concentration-dependent inhibition zones provide evidence of antibacterial activity against the tested microorganisms. However, inhibition-zone measurements are qualitative/semi-quantitative and do not directly provide the minimum inhibitory concentration (MIC) or minimum bactericidal concentration (MBC). MIC/MBC determination was not performed in the present study; therefore, no MIC or MBC values are claimed. Quantitative MIC/MBC assays would be valuable in future studies to establish the minimum concentration required for inhibition and bactericidal activity more precisely.

All quantitative measurements for which independent replicate data were available were expressed as mean ± standard deviation (SD). Error bars in the corresponding quantitative figures represent the standard deviation of the available replicate measurements, and the number of replicates and meaning of the error bars are specified in the relevant figure captions. For characterisation measurements or other datasets for which replicate measurements were not available, no artificial error estimates were introduced.

### 3.7. Reactive Oxygen Species-Mediated Antimicrobial Mechanism

**Figure 11:**
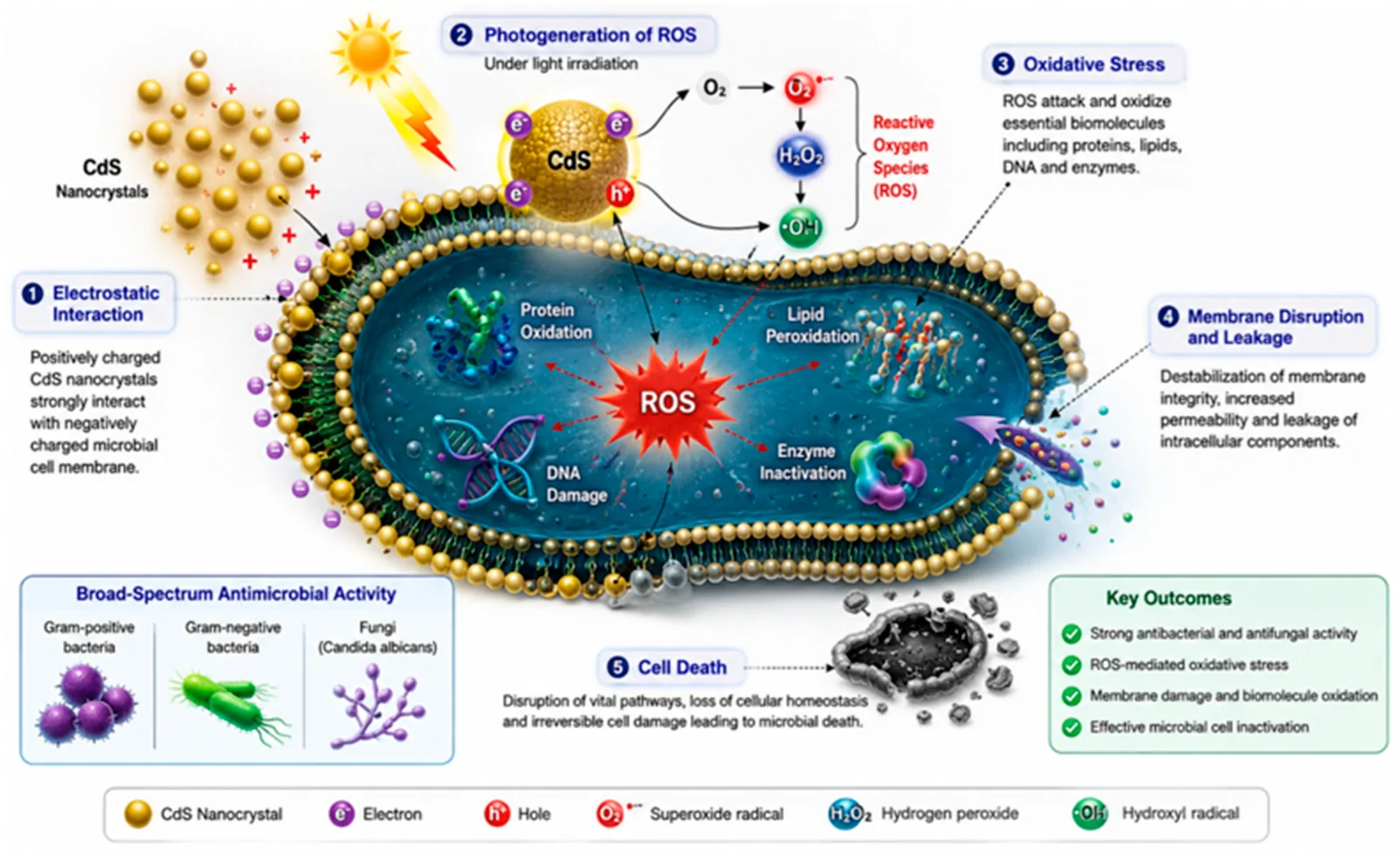
ROS-mediated antimicrobial mechanism of CdS semiconductor nanocrystals showing membrane disruption, oxidative stress, biomolecular damage, and microbial cell death under light irradiation.

The mechanism of action of the prepared CdS–SNCs against microorganisms is mainly based on ROS-induced oxidative stress along with membrane disruption effects, which are schematically presented in Figure 11. The functional antimicrobial property of the compound results from the synergistic effect of surface-active CdS nanocrystals with microbial cell membranes under physiological and light-irradiated conditions.

The ROS-based antimicrobial mechanism can be shown as follows:

$$\mathrm{CdS}+O_{2}\to O_{2}^{\cdot -}\to H_{2}O_{2}\to \cdot \mathrm{OH}$$

⋅*OH* + *Cellular biomolecules* → *Oxidized products* → *Cell death*

The comparatively higher antibacterial susceptibility of Gram-positive bacteria compared to Gram-negative bacteria can be attributed to differences in cell-wall architecture. Gram-positive bacteria have a thicker layer of peptidoglycan, which makes them very vulnerable to nanoparticle attachment and oxidative damage by ROS. However, Gram-negative bacteria have another layer of lipopolysaccharides, which to some extent prevents the penetration of nanoparticles. However, ROS molecules can pass through the pores and cause destabilisation of the outer membrane, eventually causing bacterial cell death.

Likewise, fungi like *Candida albicans*, ROS production and membrane interaction cause the destruction of ergosterol-containing membranes, mitochondrial dysfunction, inhibition of sporulation, and oxidative stress-induced damage to fungal cells. The superior antimicrobial properties of CdS–SNCs may thus be due to the synergistic action of electrostatic interaction of the membrane with the particle, ROS-induced oxidative stress, oxidation of intracellular biomolecules, and interference with crucial metabolic pathways. The wide-spectrum antimicrobial activity exhibited by the compound against bacteria and fungi clearly indicates its considerable utility as an antimicrobial agent for various applications.

Moreover, the green synthesis strategy and multifunctional antimicrobial activity of CdS–SNCs effectively endorse their significance in terms of various United Nations Sustainable Development Goals (SDGs) related to SDG-3 (Good Health and Well-being), SDG-6 (Clean Water and Sanitation), SDG-7 (Affordable and Clean Energy) and SDG-12 (Responsible Consumption and Production).

### 3.8. Comparison with Recently Reported CdS-Based Systems

The comparative performance of the current system based on CdS relative to recent research articles related to photocatalytic, electrochemical, and antibacterial properties is provided in Table 7 below. In particular, the current green synthesis method produced CdS nanoparticles with competitive visible-light-induced photocatalytic activity, narrow optical band gap, electrochemical activity, and strong antibacterial action against both Gram-positive and Gram-negative bacteria. As compared with the other heterostructure/hybrid systems based on CdS reported earlier, the current approach offers simplicity in preparation and multifunctional use of CdS semiconductor nanoparticles.

Compared with recently reported pristine and composite CdS materials, the present study provides an integrated multifunctional assessment of pristine wurtzite CdS. However, direct quantitative comparison of the reported performance values should be interpreted with caution because the literature studies were conducted under different experimental conditions. Photocatalytic performance may vary with catalyst dosage, pollutant concentration, irradiation source and intensity, reaction time, solution pH, and reactor configuration, whereas electrochemical performance depends on electrode composition, active-material loading, electrolyte, scan rate, current density, and cell configuration. Therefore, the values summarised in Table 7 are presented primarily for contextual benchmarking and should not be regarded as a direct ranking of the intrinsic superiority of the investigated CdS nanocrystals.

## 4. Conclusions

In this study, wurtzite CdS semiconductor nanocrystals (CdS–SNCs) were successfully synthesised through a simple, low-cost, and environmentally friendly co-precipitation method. XRD confirmed the formation of phase-pure hexagonal wurtzite CdS with good crystallinity, while FESEM, EDX, and HRTEM analyses supported the nanoscale morphology, elemental composition, and crystalline characteristics of the synthesised material. The CdS–SNCs exhibited an optical bandgap of 2.15 eV, supporting their visible-light-responsive behaviour. The synthesised CdS–SNCs achieved 89.93% methylene blue degradation within 120 min under visible-light irradiation, demonstrating their photocatalytic activity. Concentration-dependent antibacterial and antifungal activity was also observed against the tested microorganisms. Electrochemical measurements performed in a three-electrode configuration yielded a specific capacitance of 17.75 F g^−1^, indicating measurable electrochemical activity of the pristine CdS–SNCs.

The novelty of this work lies not in the co-precipitation synthesis route itself, but in the integrated investigation of the structural, optical, photocatalytic, electrochemical, antimicrobial, and theoretical characteristics of pristine CdS–SNCs within a single study. The DFT calculations provided qualitative insight into the electronic structure and charge distribution of the finite CdS cluster model. Importantly, the calculated HOMO–LUMO gap was not considered equivalent to the experimentally determined optical bandgap of CdS, and the DFT results were interpreted only as qualitative electronic structure descriptors.

The present study also has several limitations. The involvement of specific reactive oxygen species in photocatalytic degradation was not experimentally confirmed because ROS-scavenging or direct ROS detection experiments were not performed. In addition, two-electrode full-cell measurements and quantitative MIC/MBC assays were not conducted. Therefore, the reported electrochemical and antimicrobial results should be interpreted within the experimental configurations employed in this study. Future investigations incorporating band-edge determination, photocurrent or charge carrier measurements, ROS-scavenging studies, two-electrode device testing, and quantitative MIC/MBC assays could provide further insight into the photocatalytic mechanism, practical energy storage performance, and antimicrobial potency of CdS–SNCs.

Overall, the results determine that the synthesised CdS–SNCs possess promising multifunctional characteristics, generally in visible-light photocatalysis, electrochemical response, and antimicrobial activity, supporting their potential for further investigation in environmental and related multifunctional applications.

## Figures and Tables

**Table 1:** Crystallographic unit-cell parameters of the synthesised CdS–SNCs.

Parameter	Value
Crystal system	Hexagonal
Crystal structure	Wurtzite
Space group	P6_3_mc
JCPDS Card No.	41-1049
Lattice parameter, *a* = *b* (Å)	4.129
Lattice parameter, *c* (Å)	6.712
Cell angles, α = β (°)	90
Cell angle, γ (°)	120
*c/a* ratio	1.625
Unit-cell volume (Å^3^)	99.08

**Table 2:** Comparison of the photocatalytic performance of the synthesised CdS semiconductor nanocrystals with previously reported CdS-based photocatalysts.

Photocatalyst	Pollutant	Light Source	Degradation (%)	Time (min)	Reference
CdS–SNCs (This work)	Methylene blue	Visible light	90	120	This work
CdS thin film	Methylene blue	160 W Hg lamp	64	120	[41]
Ni–CdS thin film	Methylene blue	160 W Hg lamp	84	120	[41]
CdS nanowires	Methylene blue	Visible light	97	180	[42]
g-C_3_N_4_/CdS/BiVO_4_ ternary composite	Methylene blue	Visible light	94.5	120	[43]

**Table 3:** Performance of reusability of CdS–SNCs in the degradation of methylene blue under visible-light irradiation.

Cycle Number	Photocatalytic Efficiency (%)	Reusability Efficiency (%)
1	89.93	100
2	88.47	98.38
3	86.92	96.66
4	84.76	94.25
5	82.94	92.22

**Table 4:** Specific capacitance values of CdS–SNC electrodes at various scan rates.

Scan Rate (mV s^−1^)	Specific Capacitance (F g^−1^)
5	17.75
10	15.43
25	11.56
50	9.87
75	8.19
100	7.84

**Table 5:** Electrochemical cycling stability of CdS–SNC electrode.

Number of Cycles	Specific Capacitance (F g ^−1^)	Capacitance Retention (%)
Initial	17.75	100
200	17.12	96.45
400	16.58	93.41
600	16.03	90.31
800	15.61	87.94
1000	15.28	86.08

**Table 6:** Antibacterial activity of CdS–SNCs against selected bacterial strains.

Concentration (mg mL^−1^)	*Staphylococcus aureus* (ZOI, mm)	*Pseudomonas aeruginosa* (ZOI, mm)	*Candida albicans* (ZOI, mm)
5	10 ± 1	8 ± 1	11 ± 1
10	14 ± 1	11 ± 1	15 ± 1
20	18 ± 1	14 ± 1	20 ± 1
Standard drug (1 mg mL^−1^)	24 ± 1 (Ciprofloxacin)	21 ± 1 (Ciprofloxacin)	25 ± 1 (Clotrimazole)
Control (DMSO)	No inhibition	No inhibition	No inhibition

All antimicrobial experiments were performed in triplicate, and values are presented as mean ± standard deviation.

**Table 7:** Comparative performance analysis of the present CdS semiconductor nanocrystals with recently reported CdS-based systems for photocatalytic, electrochemical, and antimicrobial applications.

Material System	Photocatalytic Degradation Efficiency (%)	Optical Band Gap (eV)	Specific Capacitance (F g^−1^)	Antibacterial Activity	Major Findings	Ref.
CdS semiconductor nanocrystals	89.93%/120 min (MB)	2.15	17.75	*S. aureus*: 18 mm; *P. aeruginosa*: 14 mm	Eco-friendly multifunctional CdS system with photocatalytic, electrochemical, and antimicrobial performance	Present work
Green CdS nanoparticles	70–95%/60–180 min	2.2–2.5	Rarely reported	14–20 mm	Plant-mediated green synthesis with visible-light photocatalytic activity	[75]
CdS heterojunction	85–95%/90–120 min	2.1–2.4	35–120	12–18 mm	Enhanced charge separation and photocatalytic kinetics	[76]
Phyto-mediated CdS	75–92%/60–150 min	2.2–2.6	Not reported	15–22 mm	Strong ROS-mediated antimicrobial and photocatalytic behaviour	[77,78]
CdS hybrid composite	—	—	1262	Generally, not evaluated	Excellent electrochemical capacitance with hybrid architecture	[79]

## Data Availability

The data supporting the findings of this study are available from the corresponding authors upon reasonable request.

## Abbreviations

The following abbreviations are used in this manuscript:

CdS	Cadmium sulphide
CdS–SNCs	Cadmium sulphide semiconductor nanocrystals
DFT	Density functional theory
XRD	X-ray diffraction
FESEM	Field-emission scanning electron microscopy
HRTEM	High-resolution transmission electron microscopy
EDAX	Energy-dispersive X-ray analysis
AFM	Atomic force microscopy
UV–Vis	Ultraviolet–visible spectroscopy
MB	Methylene blue
ROS	Reactive oxygen species
CV	Cyclic voltammetry
GCD	Galvanostatic charge–discharge
EIS	Electrochemical impedance spectroscopy
GCE	Glassy carbon electrode
SCE	Saturated calomel electrode
PBS	Phosphate-buffered saline
KCl	Potassium chloride
Na_2_SO_4_	Sodium sulphate
DMSO	Dimethyl sulfoxide
SDA	Sabouraud dextrose agar
ZI	Zone of inhibition
GGA	Generalised gradient approximation
PBE	Perdew–Burke–Ernzerhof
DNP	Double numerical basis set with polarisation
DSPP	Density functional semi-core pseudopotential
SCF	Self-consistent field
FMO	Frontier molecular orbital
HOMO	Highest occupied molecular orbital
LUMO	Lowest unoccupied molecular orbital
CDD	Charge density difference
FWHM	Full width at half maximum
RSD	Relative standard deviation
SDGs	Sustainable Development Goals
